# Characterization of intracellular membrane structures derived from a massive expansion of endoplasmic reticulum (ER) membrane due to synthetic ER-membrane-resident polyproteins

**DOI:** 10.1093/jxb/erad364

**Published:** 2023-09-16

**Authors:** Andras Sandor, Marketa Samalova, Federica Brandizzi, Verena Kriechbaumer, Ian Moore, Mark D Fricker, Lee J Sweetlove

**Affiliations:** Department of Biology, University of Oxford, South Parks Road, Oxford, UK; Department of Experimental Biology, Masaryk University, Brno, Czech Republic; MSU-DOE Plant Research Laboratory, Department of Plant Biology, Michigan State University, East Lansing, Michigan, USA; Department of Biological and Medical Sciences, Oxford Brookes University, Oxford, UK; Department of Biology, University of Oxford, South Parks Road, Oxford, UK; Department of Biology, University of Oxford, South Parks Road, Oxford, UK; Department of Biology, University of Oxford, South Parks Road, Oxford, UK; Swedish University of Agricultural Sciences, Sweden

**Keywords:** Compartment, endoplasmic reticulum, membrane, OSER, proliferation, synthetic biology

## Abstract

The endoplasmic reticulum (ER) is a dynamic organelle that is amenable to major restructuring. Introduction of recombinant ER-membrane-resident proteins that form homo oligomers is a known method of inducing ER proliferation: interaction of the proteins with each other alters the local structure of the ER network, leading to the formation large aggregations of expanded ER, sometimes leading to the formation of organized smooth endoplasmic reticulum (OSER). However, these membrane structures formed by ER proliferation are poorly characterized and this hampers their potential development for plant synthetic biology. Here, we characterize a range of ER-derived membranous compartments in tobacco and show how the nature of the polyproteins introduced into the ER membrane affect the morphology of the final compartment. We show that a cytosol-facing oligomerization domain is an essential component for compartment formation. Using fluorescence recovery after photobleaching, we demonstrate that although the compartment retains a connection to the ER, a diffusional barrier exists to both the ER and the cytosol associated with the compartment. Using quantitative image analysis, we also show that the presence of the compartment does not disrupt the rest of the ER network. Moreover, we demonstrate that it is possible to recruit a heterologous, bacterial enzyme to the compartment, and for the enzyme to accumulate to high levels. Finally, transgenic Arabidopsis constitutively expressing the compartment-forming polyproteins grew and developed normally under standard conditions.

## Teaser

A systematic characterization of ER-derived membranous structures highlights their capacity for effective sub-cellular compartmentalization without disrupting the host.

## Introduction

The endoplasmic reticulum (ER) is a remarkably flexible and dynamic structure, sandwiched between the tonoplast and the plasma membrane in plant cells (Stefano and Brandizzi, 2018; Kriechbaumer and Brandizzi, 2020; Li *et al.*, 2022). As the organelle with the largest surface area, the ER is involved in a remarkable number of cellular functions. It plays a key role in the biosynthesis and quality control of proteins (Stefano *et al.*, 2014; Barlowe and Helenius, 2016), intracellular signalling and maintaining homeostasis, e.g. by functioning as an important element in Ca^2+^ storage (Friml and Jones, 2010; Kriechbaumer and Brandizzi, 2020), provides a surface for numerous metabolic reactions (Hawes *et al.*, 2015; Obata, 2019; Smirnoff, 2019), as well as intracellular trafficking as a core part of the secretory pathway (Kriechbaumer and Brandizzi, 2020; Li *et al.*, 2022).

The ER is a single, continuous organelle, composed of cisternae and tubules interconnected by three-way junctions (Stefano and Brandizzi, 2018; Kriechbaumer and Brandizzi, 2020). The presence of these three sub-domains of the ER are governed by three families of proteins that respectively facilitate their development: the Lunapark proteins (Kriechbaumer *et al.*, 2018), the reticulons (Voeltz *et al.*, 2006; Shibata *et al.*, 2008; Sparkes *et al.*, 2010), and atlastins (Chen *et al.*, 2011; Ueda *et al.*, 2016). In turn, the overall structure of the ER is based on the relative proportion of the three ER sub-domains (Kriechbaumer and Brandizzi, 2020). The ER structure is capable of changing rapidly, with local interconversion of cisternae and tubules happening within seconds, and larger scale structural changes occurring on the scale of hours or days (Sparkes *et al.*, 2011; Pain and Kriechbaumer, 2020). These changes can be driven by both exogenous and endogenous cues (Almsherqi *et al.*, 2009; Stefano and Brandizzi, 2018). This flexibility of structure can be linked to the various functions the ER takes part in: for example, cells with greater secretory activity (e.g. peripheral secretory cells in maize root caps (Stephenson and Hawes, 1986) tend to have higher proportions of cisternae (Shibata *et al.*, 2006; Sparkes *et al.*, 2011).

The ability of the ER to change shape allows it to take on some highly specialized forms. One of these is the expansion of the ER and the appearance of tightly stacked arrays of smooth ER sheets, forming concentrated whorls or regular sinusoidal arrays with cubic symmetry, collectively known as organized smooth endoplasmic reticulum (OSER; Snapp *et al.*, 2003; Borgese *et al.*, 2006; Sandor *et al.*, 2021). While OSERs were primarily investigated in yeast and mammalian cell lines, studies utilizing recombinant fluorescent proteins to explore the secretory pathway of plant cells have revealed much about how OSER is formed; for a detailed review on this topic see Sandor *et al.* (2021). These discoveries were mostly serendipitous, as oligomerising membrane-bound proteins often accidentally trigger the formation of OSER (Yamamoto *et al*., 1996; Snapp *et al.*, 2003; Barbante *et al.*, 2008). As such, much that was uncovered about OSER was incidental and studies were not done in a systematic manner since OSER was often not the main focus of the researchers (Sandor *et al.*, 2021). Other forms of massive ER expansion are also known, e.g. in response to ER stress and the unfolded protein response (Schuck *et al.*, 2009), but again, studies often focused on yeast and mammalian cells and their understanding in plants is limited.

A systematic study of ER membrane proliferations would also be beneficial to evaluate if there is potential for these to be developed as a tool for plant synthetic biology. This has been previously suggested for OSER, especially as a compartmentalization tool due to the membranous and organized nature of its structure (Polka *et al.*, 2016; Sandor *et al.*, 2021). Development of a synthetic membranous compartment in plants is highly desirable, as it could be superior to other compartmentalization strategies, such as using membraneless organelles or repurposing organelles (Nott *et al.*, 2015; Malhotra *et al.*, 2016; Polka *et al.*, 2016; Gao and Zhou, 2019). While a novel compartment is more difficult to engineer and requires a much thorough understanding of the underlying molecular mechanisms, it is expected to have limited crosstalk with the host background, and the presence of a membrane would facilitate control over the contents of the compartment. Although some progress has been reported in recent years in this field [e.g. by the use of the maize storage protein γ-Zein to build functionalized vesicles (Reifenrath *et al.*, 2020)], there is still no broadly usable method for the generation of synthetic compartments in plants for metabolic engineers. However, to evaluate the potential of ER membrane proliferations as a compartmentalization tool in plant cells, an in-depth characterization of such a structure and knowledge on the molecular underpinnings needed to modulate its formation seem necessary.

Here, we characterize novel ER structures formed from a range of ER membrane-resident protein constructs to assess the relationship between the constitutive domains of the massive ER proliferation-inducing protein and the structure whose formation it triggers. We show that the generated compartment has a diffusional barrier to both the ER and the bulk cytosol. Surprisingly, quantitative analysis of the dynamics and morphology of the remaining ER show that it is not significantly disrupted by the formation of the membranous structure. We discuss the similarities and differences of these compartments to established OSER structures. Finally, we discuss the potential future impacts of these findings with regards to the possible use of massive ER proliferation as a compartmentalization tool in plant cells.

## Materials and methods

### Plant husbandry and materials

Ethanol-sterilized *Arabidopsis thaliana* (ecotype Col-0) seeds were grown on MS-agar [4.33 g l^–1^ Murashige and Skoog medium (Duchefa Biochemie, Haarlem, Netherlands), 15 g l^–1^ agar] medium plates with 50 µg ml^–1^ kanamycin and 200 µg ml^–1^ cefotaxime in a growth cabinet with a 16 h light/8 h dark photoperiod (at a light intensity of 120 µmol m^–2^ s^–1^) at 20 °C. Seedlings were transplanted to soil [3:1 ratio of Sinclair Pro modular seed growing compost (Sinclair Pro, Cheshire, UK) and Sinclair pro fine vermiculite with 0.4 g l^–1^ Exemptor (ICL, Ipswich, UK) as an insecticide] after 7 d and grown in a greenhouse with a 16 h light/8 h dark photoperiod at 25 °C, with natural light supplemented to achieve light intensity of up to 200 µmol m^–2^ s^–1^ using ATTIS-7 LED grow lights (Plessey, London, UK). *Nicotiana tabacum* and *Nicotiana benthamiana* seeds were sown directly onto soil and grown in a greenhouse in identical conditions as above.

### Plant transformation

Transient transformation was performed as described previously (Sparkes *et al.*, 2006) by pelleting *Agrobacterium tumefaciens* culture after overnight growth, washing twice using a modified infiltration buffer (5mM MES pH 5.6, 5 mM MgCl_2_, 500 µM acetosyringone), and injecting *A. tumefaciens* resuspended (at OD_600_=0.05) in infiltration buffer into the abaxial side of 4–6-week-old *N. tabacum* or *N. benthamiana* leaves using a needleless syringe. When multiple protein constructs were co-expressed, every *A. tumefaciens* carrying a different construct was introduced at equal concentrations to the infiltration buffer. For cargo capture experiments, the HbpA-mCherry-SpC-his construct was introduced 7 d after the first transient transformation by the C22Y or C22Y-SpyT-his constructs to allow time for the compartments to fully form.

Stable transgenic lines of Arabidopsis were obtained through transformation using the floral dip method followed by antibiotic selection (Clough and Bent, 1998).

### Genetic construct design and molecular cloning

Prior to gene synthesis, the protein constructs were modelled using pyMOL (v2.3.4, Schrödinger LLC, New York, USA) to determine the binding orientation of the oligomerising domains. The genetic constructs coding for the polyprotein scaffolds C22Y, G22C, C22Y-SpyT-his and HbpA-mCherry-SpC-his were synthesized by Twist Bioscience (San Francisco, USA) and supplied recombined in the Gateway entry vector pTWIST-ENTR. The polyprotein scaffolds 22Y and G22 were derived from C22Y and G22C, respectively, by removing the coiled-coil domain CC-Di using PCR via Phusion High-Fidelity DNA Polymerase (Thermo Fisher Scientific, Waltham, USA) according to the manufacturer’s instructions. G-Y(cyt) was generated similarly from G22Y by removing BP22 and the ER-targeting peptide. G22Y was designed and cloned as described previously (Samalova *et al.*, 2006). Briefly, a sp-mGFP5-BP22 product of overlapping PCR was digested with *Spe*I/*Xho*I and inserted into *Spe*I/*Sal*I sites of pVKHEn6-YFP_myc_ vector (Samalova *et al.*, 2006). The gene parts used for the design of the constructs are described in detail in Supplementary Table S1. The primers used for PCR are listed in Supplementary Table S2.

Constructs in the Gateway entry plasmid pTWIST-ENTR were sub-cloned into the plant Gateway expression plasmid pK7WG2 (Karimi *et al.*, 2002) using the Gateway LR Clonase II Enzyme Mix (Thermo Fisher Scientific, Waltham, USA) according to the manufacturer’s instructions. pK7WG2 plasmids were then introduced into *Escherichia coli* OneShot Mach1 T1 Phage-Resistant Chemically Competent Cells (Thermo Fisher Scientific, Waltham, USA) according to the manufacturer’s instructions. Plasmids were then subsequently transformed into *A. tumefaciens* LBA4404 competent cells using the freeze-thaw method, as described previously (Wise *et al.*, 2006).

### Confocal laser scanning microscopy

Leaf epidermis of *A. thaliana*, *N. tabacum*, and *N. benthamiana*, and seeds and stem samples of *A. thaliana* were imaged using a Zeiss LSM880 confocal microscope equipped with an Airyscan detector (Carl Zeiss AG, Oberkochen, Germany). Images were commonly captured with a C-Apochromat 40×/1.2 W autocorrect M27 water-immersion objective (Carl Zeiss AG, Oberkochen, Germany) at 1024 × 1024 px resolution at pixel spacing of 20–100 nm with 4-line averaging and 16-bit depth at maximum speed, with typical excitation at 488 nm (GFP), 514 nm (YFP and chloroplast), or 561 nm (RFP and mCherry), and emission at 490–510 nm (GFP), 520–560 nm (YFP), 590–640 nm (RFP and mCherry) or 660–700 nm (chloroplasts). When imaging two fluorophores in one sample, care was taken to avoid spectral overlap by using sequential excitation and line switching.

For experiments focusing on quantitative analysis of the ER network and dynamics and fluorescence recovery after photobleaching (FRAP), the delay between the images in time-series was minimized by reducing image resolution to 512 × 512 px and using 2-line averaging to give a frame rate of approximately 125 ms image^–1^. When determining the proportion of the scaffold or cargo protein constructs that were localized to the synthetic ER compartment, whole cell *Z*-stack images were captured with extra care to avoid any signal saturation. For FRAP experiments, leaf epidermal samples were treated with 25 μM Latrunculin B (Sigma-Aldrich, St. Louis, USA) for 10 min at 20 °C to immobilize the ER immediately prior to imaging. Image analysis was performed in ImageJ (v1.52k) and statistical analysis in R (v3.6.1). Statistical comparison of different groups for FRAP was analysed using Student’s *t*-test.

RFP-HDEL (Shockey *et al.*, 2006), TAR2-RFP (Kriechbaumer *et al.*, 2016), Peredox-mCherry (Hung and Yellen, 2014), GFP-TIP1:1 (Nelson *et al.*, 2007), and YFP-PEX2 (Sparkes *et al.*, 2005) were used as fluorescent probes to visualize the ER lumen, ER membrane, cytosol, tonoplast, and peroxisomes, respectively. Co-localization analysis (Manders’ overlap analysis) was performed using the ImageJ Just Another Co-localisation Plugin (v2.1.4; Bolte and Cordelières, 2006) after a 3 × 3 median filter pass.

### Quantitative analysis of ER network architecture and dynamics

Short time-series Airyscan images (20 s) of the ER network were collected at the apical end of cells expressing a compartment-forming protein construct and RFP-HDEL. Cells were only imaged if the compartment was not visible, to visualize only the unmodified ER in the region-of-interest. Images underwent quality control in ImageJ (v1.52k) to remove time-series that had drifted in the *z*-axis or were blurry. Images drifting in the *x* or *y* axes were corrected (if possible) using the StackReg (v1.0) module (Thévenaz *et al.*, 1998).

The images were then processed and analysed using the AnalyzER software package (v1.1), following the protocols described in Pain *et al.* (2019). The parameters investigated are described in Supplementary Table S3. These parameters were then statistically analysed using MANOVA to correct for multiple comparisons followed by analysis of variance (ANOVA) to determine if there were statistically discriminating features (kinetic or morphological, in the tubules, cisternae or enclosed areas) between the ER of different compartment-expressing cells and cells only expressing RFP-HDEL.

### Transmission electron microscopy

Fully-expanded *N. tabacum* leaf sections were fixed, stained using zinc-iodine-osmium and embedded in resin, as described previously (Kittelmann, 2018). Sections of 90 nm were cut from the embedded samples, placed onto 200 mesh copper grids and then post-stained for 5 min with lead citrate. The stained sections were imaged using a FEI Tecnai T12 transmission electron microscope (FEI, Hillsboro, USA) operated at 120 kW. Images were captured by a GATAN OneView digital camera (AMETEK, Pleasanton, USA) and analysed in ImageJ (v1.52k).

### Phenotypic analysis of stably transformed *A. thaliana* plants

*Arabidopsis thaliana* seedlings (5-day-old) grown on MS-agar plates were imaged using a Leica M165FC (Leica Microsystems, Wetzlar, Germany) microscope equipped with a 10× air-immersion objective. YFP fluorescence was measured using the YFP filter set, with excitation at 480–520 nm and emission at 505–565 nm, with ~1 s exposure time, to avoid saturation of the fluorescence signal.

To measure leaf surface area of grown plants, 4- and 5-week-old plants were photographed from above using the digital camera of a Xiaomi Redmi 5 (Xiaomi Corporation, Beijing, China) at 3000 × 4000 px resolution. The collected images were analysed using LeafLab (v1.5), a custom image analysis software built in Matlab, developed by Mark Fricker (Department of Plant Sciences, University of Oxford, Oxford, UK, unpublished). LeafLab digitally segments the plants from the soil and tray using colour and light intensity thresholds which are manually defined. The basic properties (e.g. surface area, solidity, perimeter length, etc.) of the identified plants are then determined automatically by LeafLab. A Nikon D700 digital camera (Nikon Corporation, Tokyo, Japan) at 4256 × 2832 px resolution was used for side-by-side comparison photographs of grown plants.

To measure dry weight, 7-week-old *A. thaliana* shoots were dried in an oven at 60 °C for 4 d. Samples were weighed regularly over that period and the dry weight recorded once a stable value was achieved.

### Protein purification and western blotting

*Nicotiana benthamiana* was used for purification experiments due to its improved amenability to higher level expression of transiently introduced constructs than *N. tabacum*. Transformed leaves were harvested 7 d after infiltration and frozen in liquid nitrogen. Samples were extracted in 10× volume of extraction buffer [50 mM NaH_2_PO_4_, 150 mM NaCl, 50 mM ascorbic acid, 0.6 % (w/v) PVPP-40, 0.4 % (w/v) bovine serum albumin, 5 % (v/v) glycerol, 1 % (v/v) Tween20, 1 mini tablet of Pierce Protease Inhibitor (Thermo Fisher Scientific, Waltham, USA), pH 8.0 adjusted with NaOH] with a pinch of acid-washed sand. The lysate was sonicated for 10 min and centrifuged for 15 min at 4 °C at 4000 ×*g* to remove cell debris, sand, and unbroken cells.

Nickel-affinity purification of polyhistidine-tagged proteins was performed using Ni-NTA Agarose resin (Thermo Fisher Scientific, Waltham, USA) in gravity-flow columns. The protein samples were allowed to bind the resin for 18 h on a rotating shaker at 4 °C, then washed five times with 10 ml wash buffer [50 mM NaH_2_PO_4_, 150 mM NaCl, 10 mM imidazole, 5 % (v/v) glycerol, 1 % (v/v) Tween20, pH 8.0 adjusted with NaOH] and then bound proteins eluted using three washes of 3 ml of elution buffer (wash buffer with 250 mM imidazole).

Protein fractions were separated using 10% SDS-PAGE analysis. Following SDS-PAGE, proteins were transferred to a nitrocellulose membrane using electroblotting (100 V, 60 min) in ice-cold transfer buffer [20 % (v/v) methanol, 50 mM Tris base, 250 mM glycine, 3.5 mM SDS]. The nitrocellulose sheet was blocked using milk albumin [50 mM Tris-HCl, pH 7.5, 150 mM NaCl, 0.5 % (v/v) Tween20, 8 % (w/v) skimmed milk powder] for 60 min 20 °C on a shaker, then incubated with the primary antibody [1 µg ml^–1^ polyclonal rabbit IgG anti-mCherry primary antibody (Proteintech Europe, Manchester, UK)] overnight at 20 °C on an orbital shaker. The membrane was subsequently washed three times with Tris-buffered saline containing Tween20 [TBS-T: 50 mM Tris-HCl, pH 7.5, 150 mM NaCl, 0.5 % (v/v) Tween20] for 5 min each, then incubated with the secondary antibody [0.1 µg ml^–1^ polyclonal goat anti-rabbit IgG with a conjugated horseradish peroxidase (HRP—Sigma-Aldrich, St. Louis, USA)] for 60 min at 20 °C and washed three times with TBS-T for 5 min each. Visualization was performed using EZ-ECL Chemiluminescence Detection Kit for HRP (Biological Industries, Kibbutz Beit-Haemek, Israel), and a chemiluminescence detection system ImageQuant LAS4000 (GE Healthcare, Illinois, USA).

## Results

### Serendipitous discovery of an ER-remodelling polyprotein

**Fig. 1.**
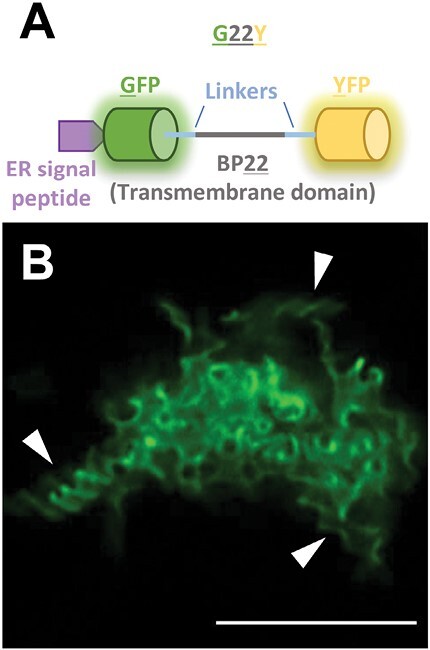
Remodelling the ER using the synthetic oligomerizing polyprotein G22Y. (A) Schematic of the G22Y polyprotein, composed of two dimerizing fluorescent proteins (GFP and YFP, facing the ER lumen and the cytosol, respectively) flanking a transmembrane domain (BP22) and a N-terminal ER targeting signal peptide. (B) Fluorescence confocal microscopy images of the structures formed in mature *N. tabacum* leaves 7 d after agroinfiltration. Arrowheads point to some notable helical organizations in the structure. The image was captured using high-resolution confocal Airyscan imaging. Scale bar=10 µm.

To interrogate trafficking in the plant endomembrane system, a fluorescent polyprotein probe was developed, consisting of a 22 amino acid transmembrane domain BP22—derived from the BP80 vacuolar sorting receptor from *Pisum sativum* (Paris *et al.*, 1997; Brandizzi *et al.*, 2002) flanked by a N-terminal GFP and a C-terminal YFP (due to this organization, the construct was named G22Y) targeted to the ER (Fig. 1A). This construct was expected to pass through the endomembrane system to the plasma membrane as it was previously reported when GFP was genetically fused to the BP22 domain (Brandizzi *et al.*, 2002). However, when transiently expressed in mature tobacco leaves, this double-tagged construct instead remained integrated into the ER forming large (up to 25 µm in diameter), self-organizing compartments as apparent from the GFP and YFP signal observed by fluorescent confocal microscopy (Fig. 1B). Notably, these G22Y structures showed a clear helical patterning (Fig. 1B).

To assess the formation of the G22Y structures, a time-course experiment was performed to image the structures every 12 h until 168 h after transient expression (Supplementary Fig. S1). The G22Y signal consistently started to appear around 36 h as small (up to 5 µm) compartments which aggregated into a single (or, in rare cases, two) large compartment per cell by 60 h. Cells with compartments remained viable up to 6 weeks, demonstrating the stability of these compartments.

**Fig. 2.**
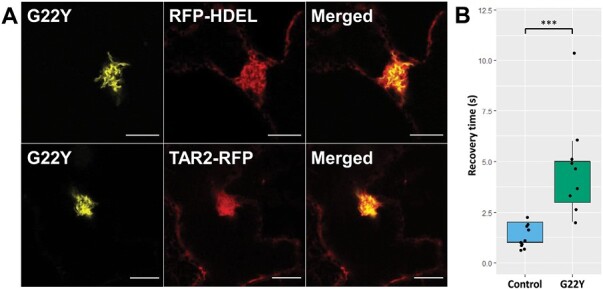
Co-expression of G22Y with fluorescent ER markers shows the connection of the compartment to the ER membrane and lumen. (A) G22Y (yellow) was co-expressed with the fluorescent ER-luminal marker protein RFP-HDEL or ER-membrane marker TAR2-RFP (both in red). The signals overlap, confirming that the G22Y compartment derives its membrane and lumen from the ER. The fluorescence confocal microscopy images were captured 7 d after agroinfiltrating mature *N. tabacum* leaves. Scale bars=10 µm. (B) The recovery rate of RFP-HDEL after FRAP of the G22Y compartment is slower when compared with the peripheral ER (Control). Asterisks depict significance levels: ***P<0.001 (Student’s *t*-test), *n*=9.

To confirm that the compartments are derived from the ER, they were co-expressed with RFP-HDEL (Nelson *et al.*, 2007) and TAR2-RFP (Kriechbaumer *et al.*, 2016), two fluorescent markers for the ER lumen and ER membrane, respectively. The overlapping signals suggested that both the lumen and the membrane of the compartment are contiguous with the ER (Fig. 2A; Supplementary Fig. S2). Co-localization was quantified using Manders’ Overlap coefficient and it was found that essentially all of the G22Y co-localized with either the RFP-HDEL lumenal marker or the TAR2-RFP membrane marker (M2=0.950 and 0.977, respectively), but there was some remaining labelled ER lumen and membrane outside the compartment (M1 =0.552 and 0.860, respectively), with more of the lumenal HDEL signal present outside the compartment (Supplementary Fig. S3). Taken together, this suggests a strong association of the construct proteins and the ER signals, but also regions of the ER devoid of G22Y. It is also notable that the cytofluorogram for the lumenal marker is quite complex and does not fit well to a linear relationship, unlike the ER membrane marker (Supplementary Fig. S3C). One possible explanation from inspection of the corresponding images is that regions with very high G22Y signal in the centre of the compartment may constrict the lumen and constrain the RFP-HDEL signal, effectively giving a decrease and then a plateau to the relationship (Supplementary Fig. S3B).

To determine if the compartment retains connection to the ER network, FRAP was used to assess lateral diffusion between the ER and the G22Y compartment, by photobleaching compartments co-expressed with RFP-HDEL or the bulk ER (control). The ER lumenal marker rapidly recovered into the compartment, confirming that it was still connected to the ER. However, the rate of recovery of fluorescence was significantly reduced when compared with the rest of the ER (*P*<0.001), suggesting a diffusional barrier between the bulk of the ER and the lumen of the G22Y compartment (Fig. 2B).

### Expanding the suite of compartment-forming polyproteins to investigate the structural basis of the ER-derived compartment

The formation of the G22Y compartment with its unusual helical organization and diffusional barrier to the lumen of the ER warranted further exploration. To determine how the protein domains affected the formation of the compartment, a range of five alternative G22Y-derived constructs were generated.

**Fig. 3.**
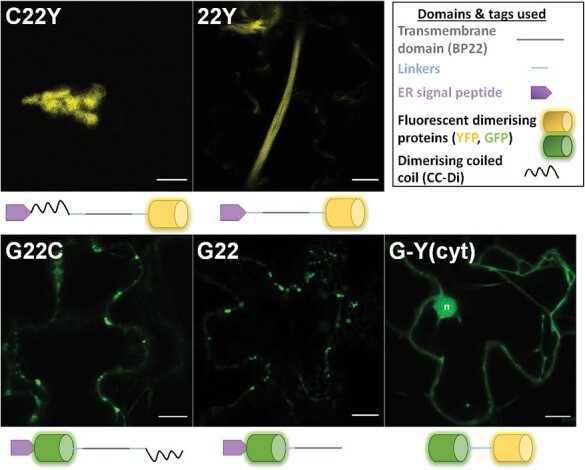
Visualizing alternative G22Y-derived constructs to expand the compartment-forming scaffold toolkit. Five new polyproteins were designed, with two constructs having either the N-terminal YFP or the C-terminal GFP removed (G22 and 22Y, respectively). In two constructs, the removed fluorescent dimerising protein was replaced with a synthetic dimerizing coiled-coil domain (G22C and C22Y), and in one construct the transmembrane domain and the ER-targeting signal peptide was removed [G-Y(cyt)]. Top right box shows the constitutive domains and the schematics of G22Y-derived constructs. Confocal microscopy images of the new constructs were taken 7 d after agroinfiltrating mature *N. tabacum* leaves. Yellow, YFP; green, GFP; n, nucleus. Scale bars=10 µm.

To assess the requirement of a cytosolic or ER-lumenal facing dimerizing domain for the formation of the compartment, we removed either of the dimerizing fluorescent proteins, to yield G22 and 22Y constructs (Fig. 3). In two further constructs (C22Y and G22Y), the removed fluorescent protein was replaced with a synthetic dimerizing coiled-coil domain CC-Di (Fletcher *et al.*, 2012), which is of similar length to GFP or YFP, but has a much stronger binding affinity (K_D_<10^–8^ M; Fletcher *et al.*, 2012) and dimerizes in parallel, in contrast to GFP and YFP, which dimerize at lower affinity (K_D_≈10^–4^ M) in an antiparallel fashion (Zacharias *et al.*, 2002). These two constructs (C22Y and G22C) enabled us to explore the effects of different binding orientations and strengths of the scaffold proteins on the compartment. The final construct G-Y(cyt) had its transmembrane domain and ER-targeting signal peptide removed to determine if ER membrane integration was essential for the formation of this compartment (Fig. 3).

Confocal fluorescence microscopy imaging of transiently transformed tobacco leaves revealed that two of the constructs formed compartments of similar (or in some cases, larger) size to the previously described G22Y compartments: 22Y often presented more elongated structures, while C22Y showed more globular compartments (Fig. 3). G22 and G22C fluorescent signals accumulated in numerous smaller (<5 µm) compartments. G-Y(cyt) did not induce the formation of any compartments and appeared to be located mainly in the cytosol and nucleus (Fig. 3). Cells with compartments showed normal cell behaviour (e.g. cytoplasmic streaming and ER network rearrangements identical to untransformed cells; Supplementary Video S1) until leaf senescence, highlighting the low apparent toxicity of the compartments in this experimental system.

To further determine the sub-cellular localization of the fluorescent signal, the new constructs were co-expressed with a range of fluorescent markers. Co-expression of C22Y and 22Y with the ER-lumenal RFP-HDEL and ER-membrane-localized TAR2-RFP revealed strong association of the two fluorescent signals, confirming that the membrane of the compartment is derived from the ER (Supplementary Figs S4, S5). The cytosolic marker Peredox-mCherry (Hung and Yellen, 2014) also partially co-localized with the C22Y and 22Y compartments, suggesting the presence of trapped cytosol in or around the compartment structure (Supplementary Figs S4, S5). These markers also highlighted the formation of several spherical vesicles in close proximity to 22Y compartments (Supplementary Fig. S6). These were labelled with both ER-membrane and ER-lumenal markers, suggesting that the construct may cause some additional disruption to the ER structure outside the main 22Y compartment. Interestingly, the membranes of these large vesicles also contained low levels of the vacuolar GFP-TIP1:1 marker, possibly caught in transit through the modified ER.

Co-expression of G22C and G22 with RFP-HDEL revealed no overlapping signal between the small compartments and the ER lumen, and the co-expression of G22C with the peroxisomal marker YFP-PEX2 revealed that the small compartments are likely peroxisomes, suggesting a mis-localization of the polyproteins instead of forming an ER-derived compartment (Supplementary Fig. S7).

### Further investigation into the C22Y and 22Y structures

**Fig. 4.**
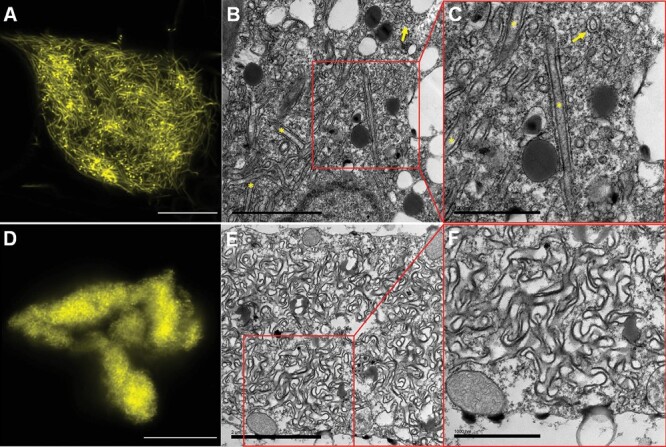
High-resolution characterization of the 22Y and C22Y compartment structures. (A–D) High-resolution Airyscan fluorescent confocal microscopy images of (A) 22Y and (D) C22Y compartments. Images were captured 7 d after agroinfiltration of mature *N. tabacum* leaves. Scale bars=10 µm. (B, C, E, F) TEM images of (B, C) 22Y and (E, F) C22Y compartments. (C) and (F) show magnified sections of (B) and (E), respectively. The 22Y structures often form two parallel membrane pairs (yellow asterisks). This is likely an ER cylinder, with some cross-sections of these highlighted with a yellow arrow. Scale bars=2 µm (B, E); 1 µm (C,F).

The two variants forming large ER-derived compartments, C22Y and 22Y, were further investigated using high-resolution airy-scanning confocal microscopy and transmission electron microscopy (TEM) to observe the detailed structures of the compartments (Fig. 4).

The two constructs induced the formation of morphologically distinct compartments. The 22Y compartments were composed of several long fibrous strands, likely double-membraned ER cylinders with cytosol trapped in the centre (Fig. 4A-C). C22Y compartments showed a more homogenous fluorescent signal, which could not be further resolved using Airyscan confocal microscopy (Fig. 4D). TEM images of C22Y compartments presented complex patterns of internal organization, with densely packed membrane structures (Fig. 4D-F), which are likely too dense for clear resolution using confocal imaging.

**Fig. 5.**
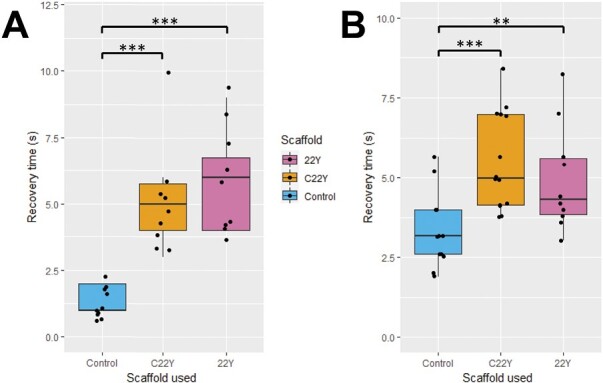
Lumenal and cytosolic fluorescence recovery after photobleaching (FRAP) rates are reduced in C22Y and 22Y compartments. FRAP experiments on the C22Y and 22Y compartment types using (A) ER lumenal RFP-HDEL and (B) cytosolic Peredox-mCherry both show a significantly increased recovery time when compared with the respective controls, suggesting a diffusional barrier between the compartment and both the ER lumen and the bulk cytosol. Asterisks depict significance levels; **P<0.01 and ***P<0.001 (Student’s *t*-test); *n*=9 for (A) and *n*=13 for (B) for all constructs. The boxes represent the interquartile range, the horizontal line in the box shows the median, and the whiskers the minimum and maximum values (excluding outliers).

To determine if the two new compartment types retain their connection to the ER, and if they have a similar diffusional barrier as the G22Y compartments (Fig. 2), FRAP was performed by co-expressing the compartment-forming proteins with RFP-HDEL. When photobleached, the RFP-HDEL signal recovered rapidly, but significantly slower than for the peripheral ER in cells not expressing compartment-inducing constructs (control) (*P*<0.001), suggesting that both the 22Y and C22Y compartments are connected to the ER, but a diffusional barrier exists (Fig. 5A).

As co-expression with Peredox-mCherry suggested that these compartments have cytosol trapped within their complex membrane structures (Supplementary Figs S2, S3), a FRAP experiment using Peredox-mCherry was also performed. This showed similar results to the RFP-HDEL FRAP, suggesting a diffusional barrier exists between the bulk cytosol and the cytosol trapped by the compartments (Fig. 5B).

The presence of the diffusional barrier to the rest of the cell suggests that molecules can be effectively compartmentalized temporarily by these structures. However, to determine if the compartment-forming scaffold proteins are also sequestered primarily in the compartment, the proportion of the total cellular fluorescence inside the compartments were measured for C22Y. This showed that the scaffold proteins almost exclusively reside in the compartment (an average of 92.7% of total cellular fluorescence was present in the compartment; Supplementary Fig. S8).

### Attaching a target protein to the compartment

To demonstrate the potential use of the compartment as a platform for accumulating recombinant proteins, we modified the C22Y scaffold to contain a C-terminal SpyTag (Reddington and Howarth, 2015). The SpyTag peptide forms a covalent bond with the cognate SpyCatcher protein (Reddington and Howarth, 2015), hence we could test whether it was possible to dock a cytosol-localized cargo protein to the compartment and for that protein to accumulate. We chose a heterologous bacterial enzyme, 2-hydroxybiphenyl-3-monooxygenase (HbpA) from the 2-hydroxbiphenyl breakdown pathway of *Pseudomonas nitroreducens*. The compound 2-hydroxybiphenyl is a fungicide, principally used for waxy citrus fruits. HpbA is an enzyme of interest for biotechnology: it has a broad substrate specificity allowing it catalyse the ortho-hydroxylation of a wide range of 2-substituted phenols to their corresponding 3’-catechols, many of which have pharmacological properties (Held *et al.*, 1998). Accordingly, we generated a construct that encoded a fusion of HbpA with mCherry (for visualization), followed by SpyCatcher to attach to the compartment-localized SpyTag.

**Fig. 6.**
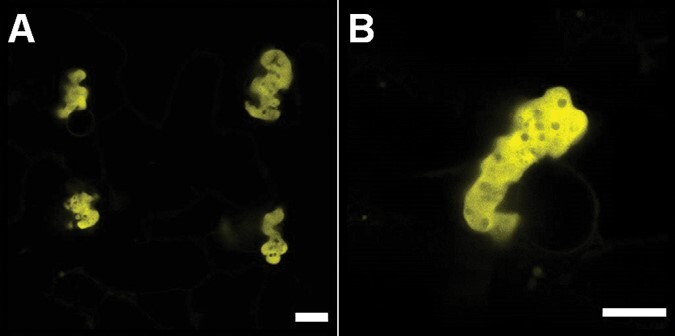
Confocal microscopy of the C22Y-SpyT-his construct. (A) Fluorescent confocal microscopy image showing four C22Y-SpyT-his compartments forming in adjacent cells. (B) High-resolution Airyscan fluorescent confocal microscopy image. Compartments formed are similar to C22Y. Images were captured 7 d after agroinfiltration of mature *N. tabacum* leaves. Scale bars=10 µm.

**Fig. 7.**
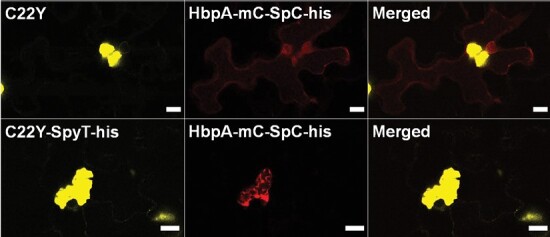
Visualization of proteins anchored to the cytosolic surface of the compartment via SpyCatcher—SpyTag covalent binding. Fluorescent confocal microscopy images (red,mCherry fluorescence; yellow, YFP fluorescence) of HbpA-mC-SpC-his with C22Y/C22Y-SpyT-his in *A. tumefaciens*-mediated transiently transformed mature *N. benthamiana* leaves. Scale bars=10 μm.

To confirm that the addition of SpyTag to C22Y did not interfere with its ability to induce the formation of a compartment, we first transiently expressed C22Y-SpyTag alone and compared it with C22Y (Fig. 6). The compartments formed were similar. Subsequently, 7 d later, leaves expressing either C22Y-SpyTag-his or C22Y were super-transformed (transiently) with the HbpA-mCherry-SpyCatcher construct, which was visualized a further 7 d later. It can be seen that in the presence of the C22Y compartment without the SpyTag, the HbpA-mCherry-SpyCatcher construct showed little overlap with the C22Y compartment and was likely cytosolic (Fig. 7). In contrast, the presence of the C22Y-SpyTag-his compartment led to relocalization of the HbpA-mCherry-SpyCatcher protein from being evenly distributed in the cytosol to being predominantly associated with the compartment. Note that this result also confirmed the expected topology of the C22Y-SpyTag-his scaffold, since it would not be possible to recruit a cytosol-targeted SpyCatcher-containing protein to the compartment, unless the SpyTag faced the cytosol (Fig. 7). Quantification of the proportion of total cellular mCherry fluorescence localized to the compartments showed that the difference was statistically significant (*P*=1.3 × 10^–16^, mean with C22Y=12.0%, mean with C22Y-SpyT-his=87.0%; Supplementary Fig. S9).

**Fig. 8.**
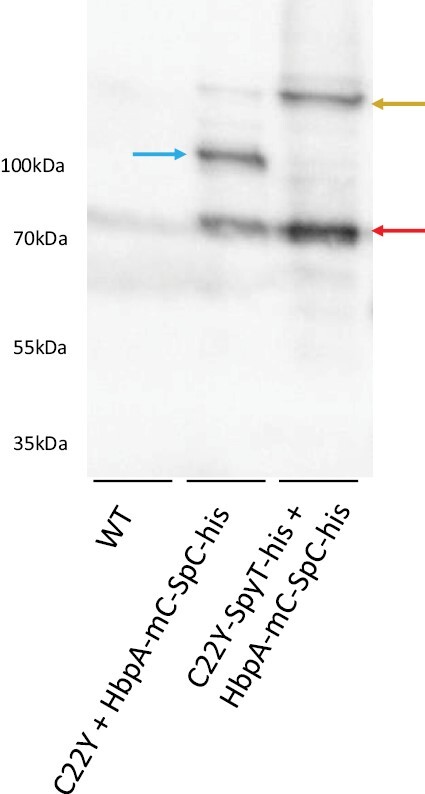
Purification of a covalently bound enzyme—protein-forming scaffold complex. Anti-mCherry western blot of elution fractions from a nickel-affinity purification from transiently transformed *N. benthamiana* samples. WT column contains untransformed plant samples, while C22Y + HbpA-mC-SpC-his and C22Y-SpyT-his + HbpA-mC-SpC-his contain plant samples co-transformed with the appropriate constructs. Blue arrow shows a band with matching molecular weight to unbound HbpA-mC-SpC-his cargo constructs (107 kDa). Golden arrow shows a band with matching molecular weight to bound HbpA-mC-SpC-his + C22Y-SpyT-his complexes (147 kDa). Red band shows putative cleavage products. The text on the left shows standard molecular weights.

The mCherry signal suggested that it is possible to recruit a cargo protein to high levels to the cytosolic face of the compartment. However, it is conceivable that the HbpA-mCherry-SpyCatcher polyprotein could have been cleaved and we were just observing the accumulation of mCherry-SpyCatcher. To investigate this and to provide an orthologous method of assessment of protein accumulation, we extracted leaves expressing the protein combinations shown in Fig. 7 and used western blotting with an anti-mCherry antibody. We were unable to detect a signal from crude extracts. We therefore took advantage of a C-terminal His tag to purify the HbpA-mCherry-SpyCatcher protein and probed western blots of the purified HbpA-mCherry-SpyCatcher fraction with an anti-mCherry antibody (Fig. 8). The results revealed that when purified from a leaf containing C22Y, an anti-mCherry-reactive band appeared at a molecular weight consistent with the complete HbpA-mCherry-SpyCatcher protein (expected molecular weight of 107 kDa). When purified from a leaf containing C22Y-SpyTag-his, the HbpA-mCherry-SpyCatcher band at ~104 kDa disappeared and a higher molecular weight band appeared at approximately the correct weight for C22Y-SpyTag-His-SpyCatcher-mCherry-HbpA (expected molecular weight of 147 kDa). A lower molecular weight band of ~70 kDa also appeared in both extracts. This was presumably a cleavage product within the Hbpa protein (61 kDa) that was still associated with mCherry (28 kDa) and the His tag (0.8 kDa). Although it is not possible to make precise quantitative conclusions from this blot, it does appear possible to recruit a similar level of HbpA protein to the compartment as can accumulate in the cytosol.

### Quantitative analysis of the architecture and dynamics of the ER

As the G22Y, C22Y, and 22Y compartments are derived from the ER, their substantial size raised the possibility of a significant disruption to the ER in the host cell. To determine if this was the case, short (20 s) time-series of cell images expressing G22Y, C22Y or 22Y co-infiltrated with the ER marker RFP-HDEL, were captured using high-resolution Airyscan confocal microscopy, focusing only on the peripheral ER (the compartments were not imaged), and quantitatively analysed using the AnalyzER software package (Pain *et al.*, 2019). During analysis, 41 parameters were measured and analysed including topological, morphological, and kinetic parameters of the ER cisternae, tubules, and the polygonal regions enclosed by them (Supplementary Table S3). ANOVAs of the 41 parameters showed that only a single one of the parameters (the average persistency of cisternae) was significantly different between the wild type (WT) and any of the compartment types (Supplementary Table S3), but this significance disappeared when accounting for multiple testing. This suggests that the formation of the compartments did not significantly disrupt the structure and dynamics of the rest of the ER in the compartment-carrying cells.

### Expressing the compartment in stably transformed *A. thaliana*

**Fig. 9.**
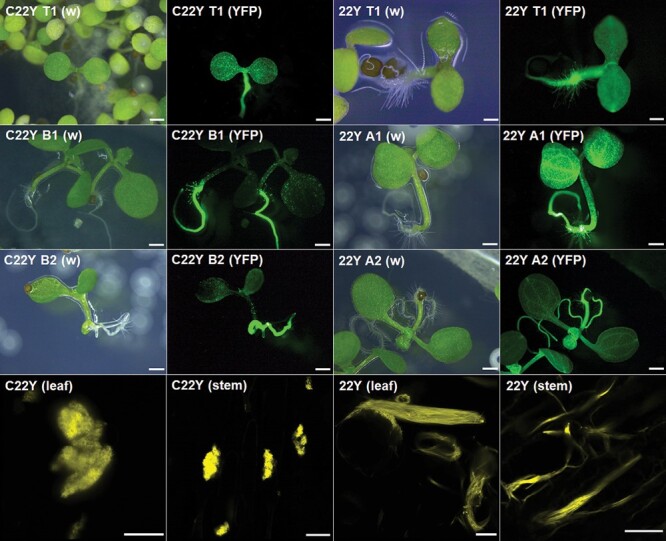
Stably transformed *A. thaliana* lines form C22Y and 22Y compartment structures similar to those obtained by transient expression in tobacco. Top three rows: fluorescent whole seedling images of 5–6-day-old stably transformed *A. thaliana* on MS media plates. Pairs of images show the white light (w) and YFP fluorescence (YFP) of T_1_ (top row) and T_2_ (second and third row) plants. A1, A2, and B1, B2 refer to two independent lines of 22Y- and C22Y-expressing *A. thaliana* lines, respectively. Fluorescent signal is present in all tissues but is noticeably stronger in the roots of T_2_ plants. Scale bars=1 cm. Bottom row: high-resolution Airyscan laser confocal microscopy images of leaf epidermis and stem cross-section of C22Y and 22Y expressing 6-week-old T_2_ plants. Both compartment types show identical morphology to the previously described compartments following transient expression of each construct. Scale bars=10 µm.

To determine if the compartments can be developed and maintained in stably transformed *A. thaliana* as well as with transient expression, the two constructs which produced the largest compartments, C22Y and 22Y, were introduced under the drive of the strong constitutive cauliflower mosaic virus *35S* promoter (Odell *et al.*, 1985). T_1_ seedlings were selected based on antibiotic resistance and inspected for fluorescence, and two independent T_2_ lines were isolated for each construct (Fig. 9). Seedlings showed YFP fluorescence in all tissue types, but T_2_ seedlings had noticeably stronger signal in their roots.

Six-week-old T_2_*A. thaliana* plants were imaged using fluorescence confocal microscopy to determine if compartments formed, and if their morphologies were identical to what was observed in transiently transformed tobacco epidermis. The stably transformed plants showed fluorescence in all cells in the stem and leaves, and fluorescent signal was detected in seeds as well (Supplementary Fig. S10). High-resolution Airyscan confocal microscopy images of the stem and leaves of stably transformed *A. thaliana* plants confirmed that the C22Y and 22Y compartments have a phenotype consistent with the transiently expressed compartments described above (Fig. 9).

Following confirmation of the presence of the C22Y and 22Y compartments, the macro-phenotypes of the stable lines were characterized using the LeafLab software package (Mark Fricker, unpublished) (Supplementary Fig. 11A, B). C22Y lines showed accelerated growth when compared with WT or 22Y, including quicker growth of the leaves, and the appearance of flowering spike growth a week before WT lines. The leaf growth rates of 22Y and WT were similar, but 22Y lines showed consistent delays in flowering spike growth (Supplementary Fig. 11C).

LeafLab was used to measure leaf area, leaf convex area, and solidity of 4- and 5-week-old seedlings (Supplementary Fig. S11B) to gain insight into the photosynthetic capacity, and shape and spread of leaf coverage of the stably modified plants. Statistical analysis of leaf area (Supplementary Fig. S12 A, B) showed that C22Y and 22Y lines had significantly greater leaf surface area than WT at weeks 4 and 5. Leaf convex area was greater for three out of four lines (with the exception of 22Y A1) when compared with WT. Solidity of leaf coverage was greater for the quicker growing C22Y lines at week 4, but this was overtaken by the other lines by week 5, with 22Y A1 showing the most even coverage (Supplementary Fig. S12A-B; Supplementary Table S4).

Above-ground dry weight of the plants was measured at week 8, with 22Y A1 and C22Y B2 showing significantly lower values than WT. These results were similar to that of stem and seed pod dry weight, which is the source of most of the weight at this timepoint for Arabidopsis. Leaf dry weight was lower for the C22Y B1 line and higher for both 22Y lines (Supplementary Fig. S12C; Supplementary Table S4). This was in line with visual inspection, with 22Y lines having more green leaves at week 8 than other lines, likely due to the delayed development of the flowering spike.

## Discussion

Building subcellular compartments is one of the central challenges of synthetic biology due to the numerous applications that require separation of functions from the bulk of the cell. Here we have shown that massive ER-membrane proliferation, which was previously primarily viewed as a disruption of ER morphology, can be used as a viable tool for this purpose in plants. The structures described above had a diffusional barrier to the bulk of the cell and the lumen of the ER, providing effective compartmentalization, without significantly disrupting the rest of the ER.

### Effects of the dimerising domain topology on compartment structure

From our experiments it is clear that the changes made to the dimerizing domains used in the synthetic polyprotein constructs have a great impact on the observed compartment morphology. In our study, a cytosol-facing antiparallel dimerising domain was essential, as removal or replacement of it with a parallel binding domain led to mislocalization to the peroxisomes, instead of the formation of large ER-derived compartments.

The ER lumenal dimerising domain also had a clear effect on the compartment morphology, since its modification (from GFP to dimerising coiled-coil domain or complete removal) led to two compartments with structures completely different from the G22Y compartments (Figs 3, 4). It is possible that the binding topology (antiparallel, parallel, or missing) has an effect on which membranes can be bridged by these polyprotein oligomers, leading to these differences. An antiparallel binding domain is expected to be more flexible, capable of bridging different membranes together in *trans*, while a parallel binding domain is spatially more restricted, likely to only bind to the same membrane in *cis*. Further studies using more compartment-forming polyproteins could be used to gain more insight into the exact relationship between the oligomerizing domain topologies and the induced compartment structures.

### Comparison with previously described plant OSER structures

Similarities between the synthetic compartments and previously described OSER structures are notable enough to warrant comparison. Both classes are derived from the ER, forming large membranous structures which retain some connection to the ER. Indeed previous FRAP experiments on OSER also showed a reduced diffusion between the ER lumen and the OSER structure (Marti *et al.*, 2010), similar to our findings in this study (Fig. 2B). Furthermore, the formation of OSER did not affect the normal functioning of the ER (Ferrero *et al.*, 2015), and the quantitative analysis of the ER network in our study (Supplementary Table S3) also showed no significant disturbance to the ER. Plants stably transformed with the compartments developed reasonably normally, similar to previous studies for OSER (Ferrero *et al.*, 2015).

Morphologically, the C22Y compartments are also similar to some of the larger OSER structures (Ferrero *et al.*, 2015; Sandor *et al.*, 2021), and the fibrous 22Y structures resemble elongated lamellae, another typical OSER phenotype (Sandor *et al.*, 2021). Notably, 22Y compartments are morphologically very similar to the OSER structures described that are induced by the *nuc* mutation of the MVP1 (modified vacuole phenotype) protein in *A. thaliana* (Jancowski *et al.*, 2014). However, MVP1 is predicted to be a non-membrane resident monomeric protein indirectly involved in ER-to-Golgi export, and the mutation was hypothesized to induce OSER by blocking this export pathway, inadvertently causing the accumulation of other OSER-inducing proteins in the ER (Jancowski *et al.*, 2014). In contrast, in this study, the results from the quantitative ER analysis using AnalyzER showed no major disruptions to the ER (Supplementary Table S1), making it unlikely that 22Y uses the same method to cause the formation of the compartment described in this study.

Equally, there are also clear morphological differences between the compartments described here and OSER. Indeed, to our knowledge, the helical patterning of G22Y compartments has not been described previously in connection with OSER. OSER also usually presents as multiple structures in a cell, unlike in our study, where a single compartment was formed almost exclusively in each cell. Furthermore, in the TEM images of 22Y (Fig. 4) some ribosomes were clearly visible along the double membranes of the tubular structure. However, whether OSER structures are exclusively composed of smooth ER is a subject of discussion, with some evidence suggesting that these ER structures can form rough ER as well (Cresti *et al.*, 1985; Fasana *et al.*, 2010; Sandor *et al.*, 2021).

The induction of compartment formation also presents some similarities to OSER formation. OSER can be induced by two types of proteins: ER-resident membrane proteins and proteins disrupting ER-to-Golgi export (Sandor *et al.*, 2021). For the former group, there is some controversy in the literature to the necessity of a cytosol-facing oligomerizing domain, with some studies showing it to be essential (Yamamoto *et al*., 1996; Snapp *et al.*, 2003; Barbante *et al.*, 2008), while others refuting this claim (Marti *et al.*, 2010; Jancowski *et al.*, 2014; Ferrero *et al.*, 2015; Grados-Torrez *et al.*, 2021). Recently, these opposing views were attempted to be reconciled into a single unified theory of OSER formation, which hypothesized multiple parallel pathways to induce OSER (Sandor *et al.*, 2021). In this study, only ER-resident membrane proteins were capable of inducing the formation of our compartment. Furthermore, a cytosol-facing antiparallell dimerizing domain was essential for the formation of the compartments.

Overall, this compartment system seems to have significant similarity to previously described OSER structures in terms of formation and effects on the host cell, but there are some morphological differences as well. It is currently unclear if the structures described in this study could be classified as a sub-group of OSER, and further comparative investigation is warranted to answer this question.

### Potential applications of the synthetic compartment system

The compartment system described in our study has a number of valuable properties that make it a good candidate for functionalization as a plant synthetic biology tool. No detrimental effects were observed in transiently or stably transformed plants, although it should be noted that an exhaustive phenotypic analysis of stably transformed plants under a range of environment conditions was not performed. There is undoubtedly a cost to bearing such a substantial expansion of the endomembrane system in every cell of the plant, and it may well be that under conditions that place the plant under energy stress or carbon limitation, this could lead to developmental phenotypes. However, our quantitative analysis of the ER of transformed cells in tobacco leaves showed that the rest of the ER network was not significantly disrupted (Supplementary Table S1), and might therefore be expected to function as normal. The ER-derived compartments show a consistent morphology across two different species, and the constitutive polyprotein scaffold almost exclusively accumulates inside the compartment structure. These properties together suggest that this compartment system could be used to improve the production of recombinant proteins, especially difficult-to-express membrane proteins or toxic proteins which would be mostly sequestered from the bulk of the cell. We have demonstrated that proteins of interest can be associated with the compartment using the SpyCatcher—SpyTag system (Reddington and Howarth, 2015) to covalently bind a target recombinant protein to the scaffold.

Another potential application is to develop this system as a synthetic metabolic microdomain. This is enabled by the high specificity of the scaffold molecules to the compartment, in combination with the diffusional barrier between the compartment lumen and the ER lumen, as well as the trapped cytosol and the bulk cytosol. Together, these properties are expected to be sufficient to enable probabilistic metabolic channelling (Sweetlove and Fernie, 2018). This would be a valuable tool for the metabolic engineering of plants, by providing a way to channel metabolic flux in a pathway of interest, especially if the intermediate is unstable, toxic, or normally secreted (Sweetlove and Fernie, 2013; Cárdenas *et al.*, 2019). Indeed, similar approaches to utilize proliferated ER membranes for the production of membrane proteins or valuable metabolites has been demonstrated in yeast (Guerfal *et al.*, 2013; Arendt *et al.*, 2017), reinforcing the case for the value of this method to be developed in plants.

A new modular compartmentalization system would be of great interest for plant synthetic biology, and an ER-based membranous compartment has numerous advantages. Compared with current popular approaches (co-opting existing organelles, building novel compartments and membraneless organelles) this compartment system has a number of attractive properties: unlike membraneless organelles, it is delimited by a membrane bilayer allowing greater control over the internal composition, and is induced by a single polyprotein construct, making it similarly simple to use. While the compartment retains some connections to the ER, there is a clear diffusional barrier, making it a more specific tool rather than simply targeting proteins to the ER; it also retains some of the benefits of a novel compartment, and the host ER is not significantly impacted. Overall, these properties give this strategy notable potential as a tool for plant synthetic biology, and further experimental validation of this system would be of great value to the field.

## Supplementary data

The following supplementary data are available at *JXB* online.

Fig. S1. Expression time-course of the G22Y compartments.

Fig. S2. ER probe controls showing normal ER phenotypes.

Fig. S3. Co-localization analysis of ER-markers (RFP-HDEL and TAR2-RFP) with the G22Y compartment.

Fig. S4. Co-expression of fluorescent markers with C22Y.

Fig. S5. Co-expression of fluorescent markers with 22Y.

Fig. S6. Characterization of the 22Y spheres.

Fig. S7. Co-expression of fluorescent markers with G22C and G22.

Fig. S8. Proportion of total cellular fluorescence present in the C22Y compartment.

Fig. S9. Efficient recruitment of proteins to the cytosolic surface of the compartment via SpyCatcher—SpyTag covalent binding.

Fig. S10. Confocal microscopy images of 6-week-old stably transformed T_2_*A. thaliana* lines.

Fig. S11. Phenotypes of stably transformed *A. thaliana* T_2_ lines.

Fig. S12. Statistical analysis of key macro-phenotypes of stably transformed *A. thaliana* lines.

Video S1. ER rearrangements in a cell with a compartment.

Table S1. Gene parts used for the design of the genetic constructs.

Table S2. PCR and sequencing primers used to generate and confirm constructs.

Table S3. Parameters used in the AnalyzER software package to investigate ER network dynamics.

Table S4. Results of statistical analyses of key macro-scale phenotypes.

erad364_suppl_Supplementary_Figures_S1-S12_Tables_S1-S4

erad364_suppl_Supplementary_Video_S1

## Data Availability

All data associated with this paper are provided within the figures and supplementary data published online.

## Abbreviations

ER: endoplasmic reticulum
FRAP: fluorescence recovery after photobleaching
OSER: organized smooth endoplasmic reticulum
TEM: transmission electron microscopy
