# Supplementary material for: Characterization of intracellular membrane structures derived from a massive expansion of endoplasmic reticulum (ER) membrane due to synthetic ER-membrane-resident polyproteins
Source: J Exp Bot. 2023 Sep 16;75(1):45–59. doi: 10.1093/jxb/erad364 (PMC10735356; doi:10.1093/jxb/erad364)
Supplement: erad364_suppl_Supplementary_Figures_S1-S12_Tables_S1-S4 [file erad364_suppl_supplementary_figures_s1-s12_tables_s1-s4.pdf]

Supplementary Data

Supplementary Figure S1: Expression time-course of the G22Y compartments.

Supplementary Figure S2: ER probe controls showing normal ER phenotypes.

Supplementary Figure S3: Co-localisation analysis of ER-markers (RFP-HDEL and TAR2-RFP) with the G22Y compartment.

Supplementary Figure S4: Co-expression of fluorescent markers with C22Y.

Supplementary Figure S5: Co-expression of fluorescent markers with 22Y.

Supplementary Figure S6: Characterisation of the 22Y spheres.

Supplementary Figure S7: Co-expression of fluorescent markers with G22C and G22.

Supplementary Figure S8: Proportion of total cellular fluorescence present in the C22Y compartment.

Supplementary Figure S9: Efficient recruitment of proteins to the cytosolic surface of the compartment via SpyCatcher – SpyTag covalent binding.

Supplementary Figure S10: Confocal microscopy images of 6 weeks old stably transformed T2 *A. thaliana* lines.

Supplementary Figure S11: Phenotypes of stably transformed *A. thaliana* T2 lines.

Supplementary Figure S12: Statistical analysis of key macro-phenotypes of stably transformed *A. thaliana* lines.

Supplementary Video SV1: ER rearrangements in a cell with a compartment.

Supplementary Table S1: Parameters used in the AnalyzER software package to investigate ER network dynamics.

Supplementary Table S2: Results of statistical analyses of key macro-scale phenotypes.

Supplementary Table S3: Gene parts used for the design of the genetic constructs.

Supplementary Table S4: PCR and sequencing primers used to generate and confirm constructs.

Supplementary Materials

Supplementary Figure S1 (below): Expression time-course of the G22Y compartments. Fluorescent confocal microscopy images of transiently transformed mature *N. tabacum* leaves shows how several smaller compartments aggregate into one or two large compartments per cell around 48-60 h after agroinfiltration. No expression of G22Y was detected before 24 h. Images shown here were collected from the same plant over the course of a week, and is representative of the morphologies and progression of appearance of compartments. In some cases, fluorescence in compartments was seen up to 6 weeks. Scale bars: 10 µm.

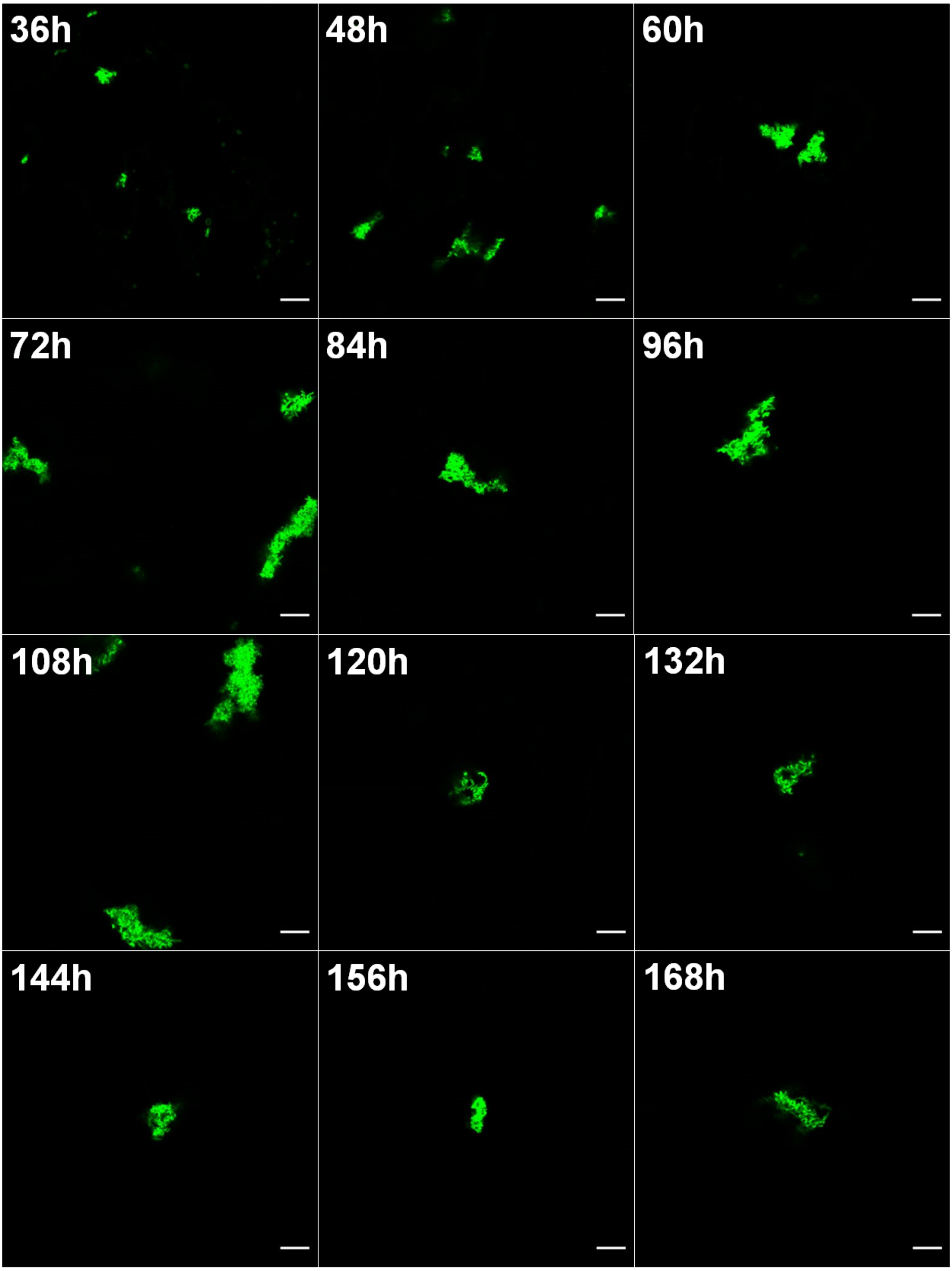
36h
48h
60h
72h
84h
96h
108h
120h
132h
144h
156h
168h

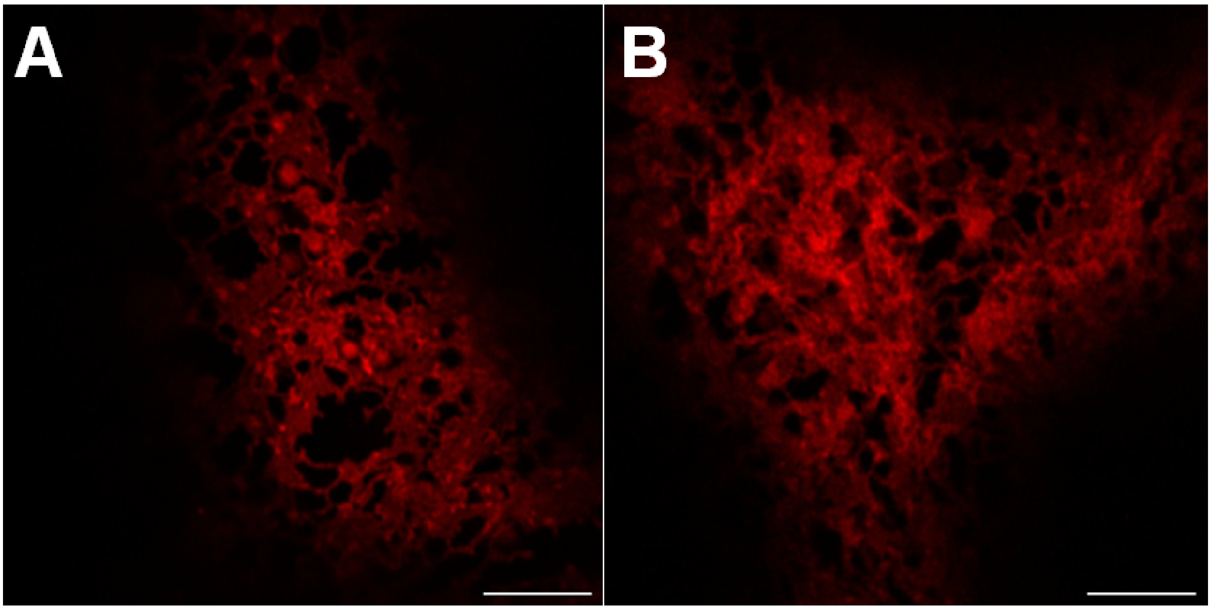

Supplementary Figure S2: ER probe controls showing normal ER phenotypes. RFP-HDEL (A) and TAR2-RFP (B) were transiently expressed in mature *N. tabacum* leaves as a control to show the probes produce the expected ER phenotypes. Scale bars: 5 µm.

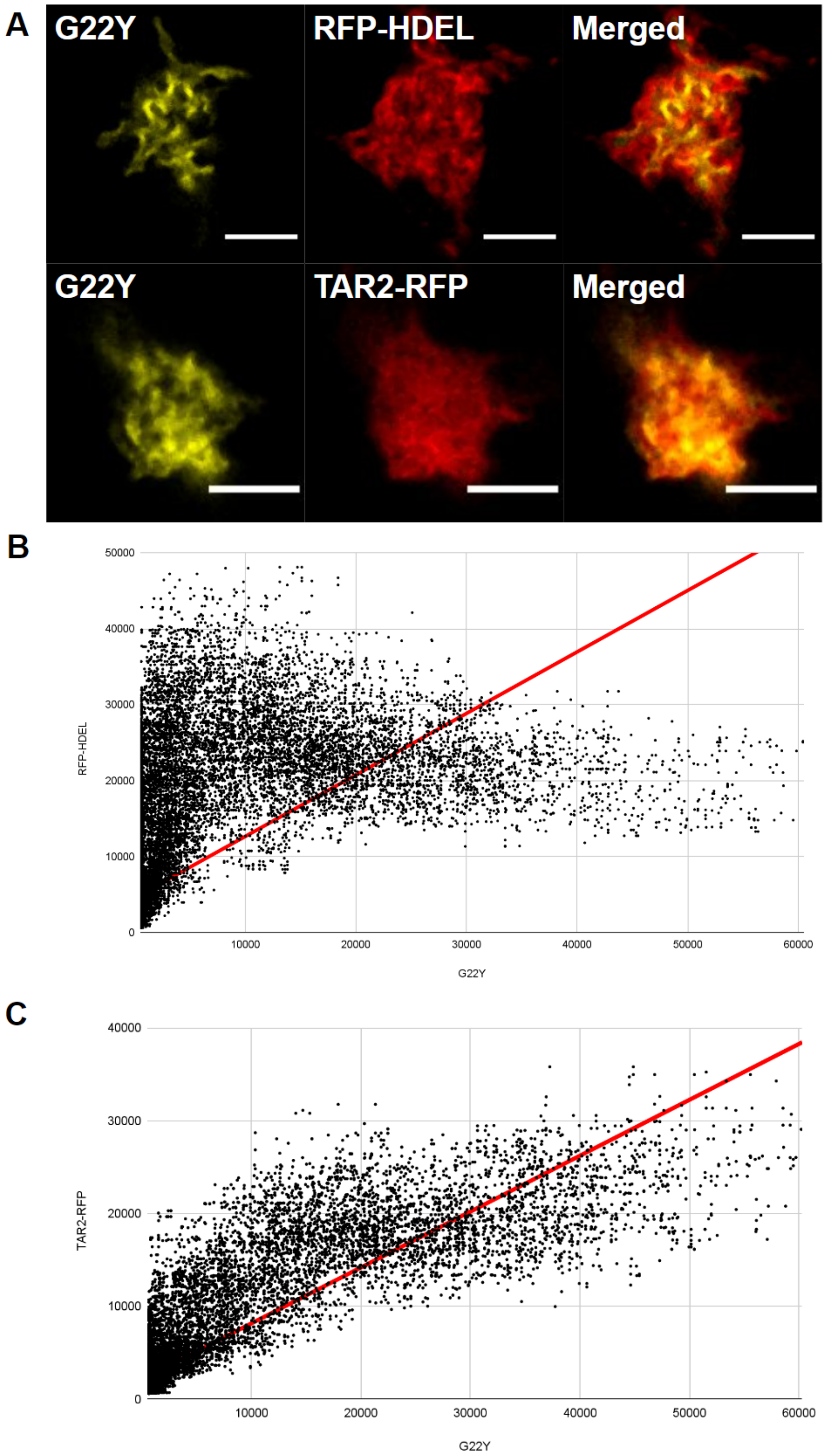

Supplementary Figure S3: Co-localisation analysis of ER-markers (RFP-HDEL and TAR2-RFP) with the G22Y compartment. A: Cropped images from Figure 2, showing the compartments. The images were de-noised using a 3x3 median filter before analysis. Scale bars: 5 µm. B and C: Cytofluorograms from the co-localisation analysis using JaCoP.

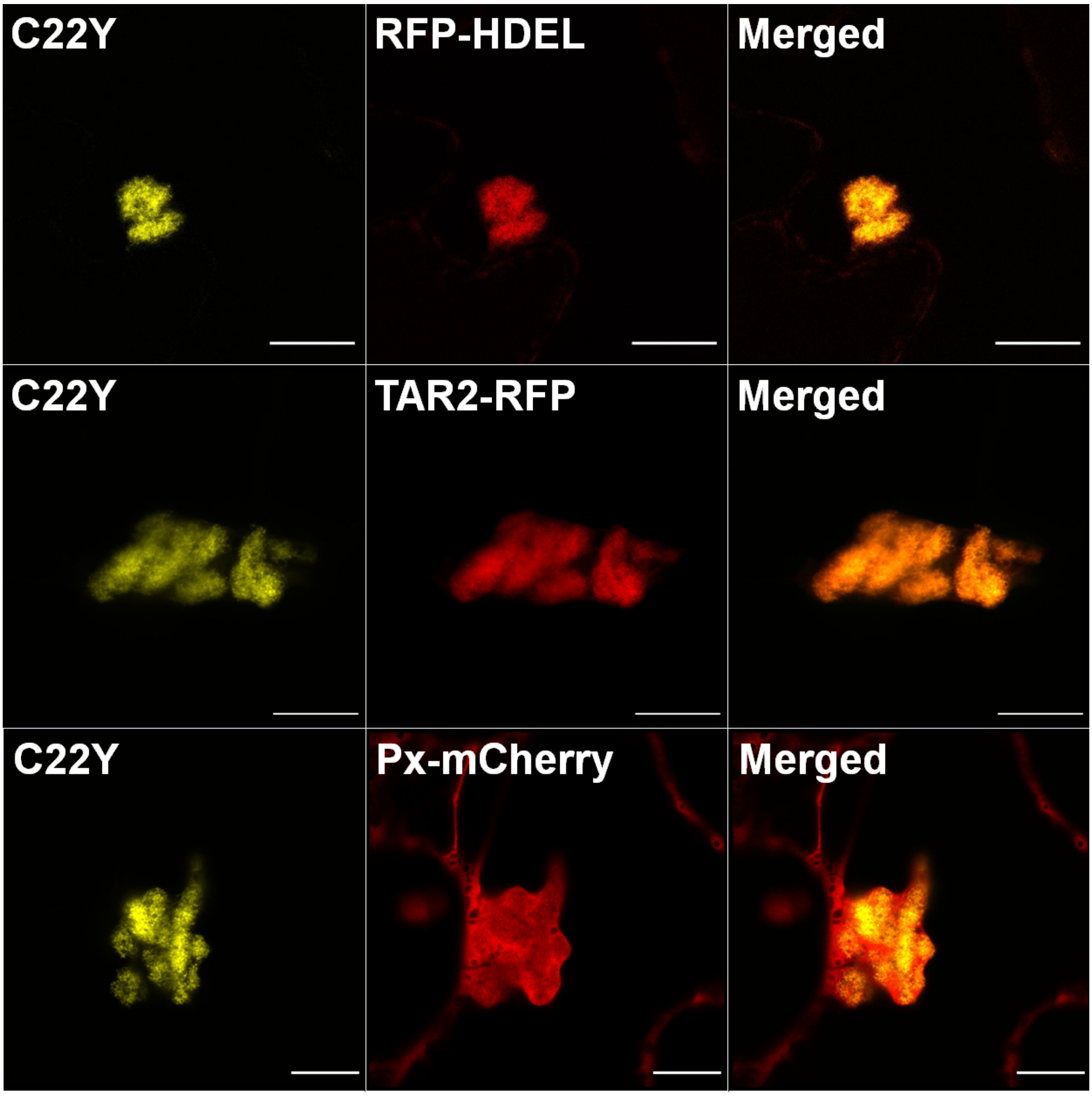

Supplementary Figure S4: Co-expression of fluorescent markers with C22Y. The three fluorescent markers RFP-HDEL (ER lumen), TAR2-RFP (ER membrane) and Peredox-mCherry (cytosol) all show association with the C22Y signal. Images were captured 7 days after agroinfiltration of mature *N. tabacum* leaves. Scale bars: 10 µm.

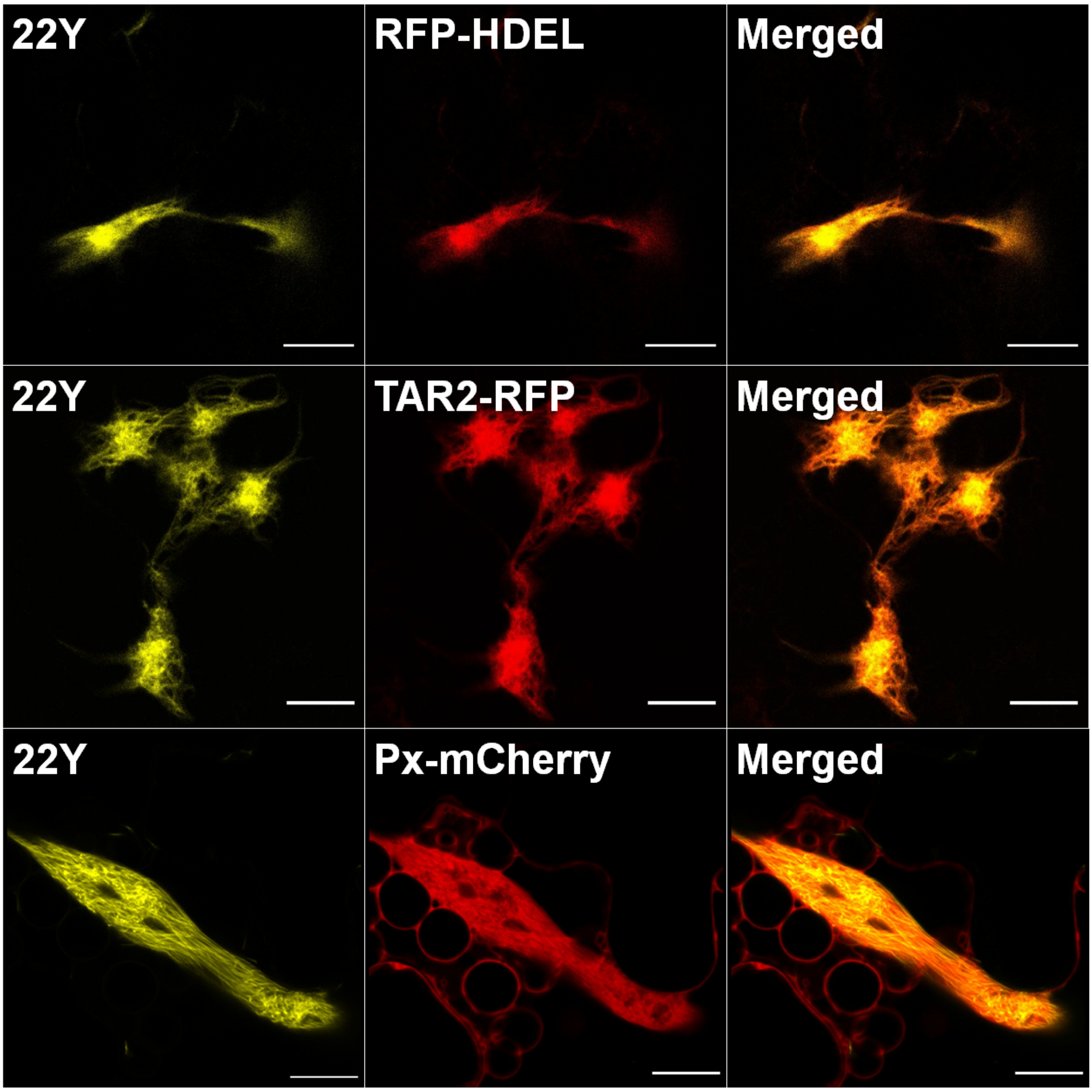

Supplementary Figure S5: Co-expression of fluorescent markers with 22Y. The three fluorescent markers RFP-HDEL (ER lumen), TAR2-RFP (ER membrane) and Peredox-mCherry (cytosol) all show co-localisation with the 22Y signal. Images were captured 7 days after agroinfiltration of mature *N. tabacum* leaves. Scale bars: 10 µm.

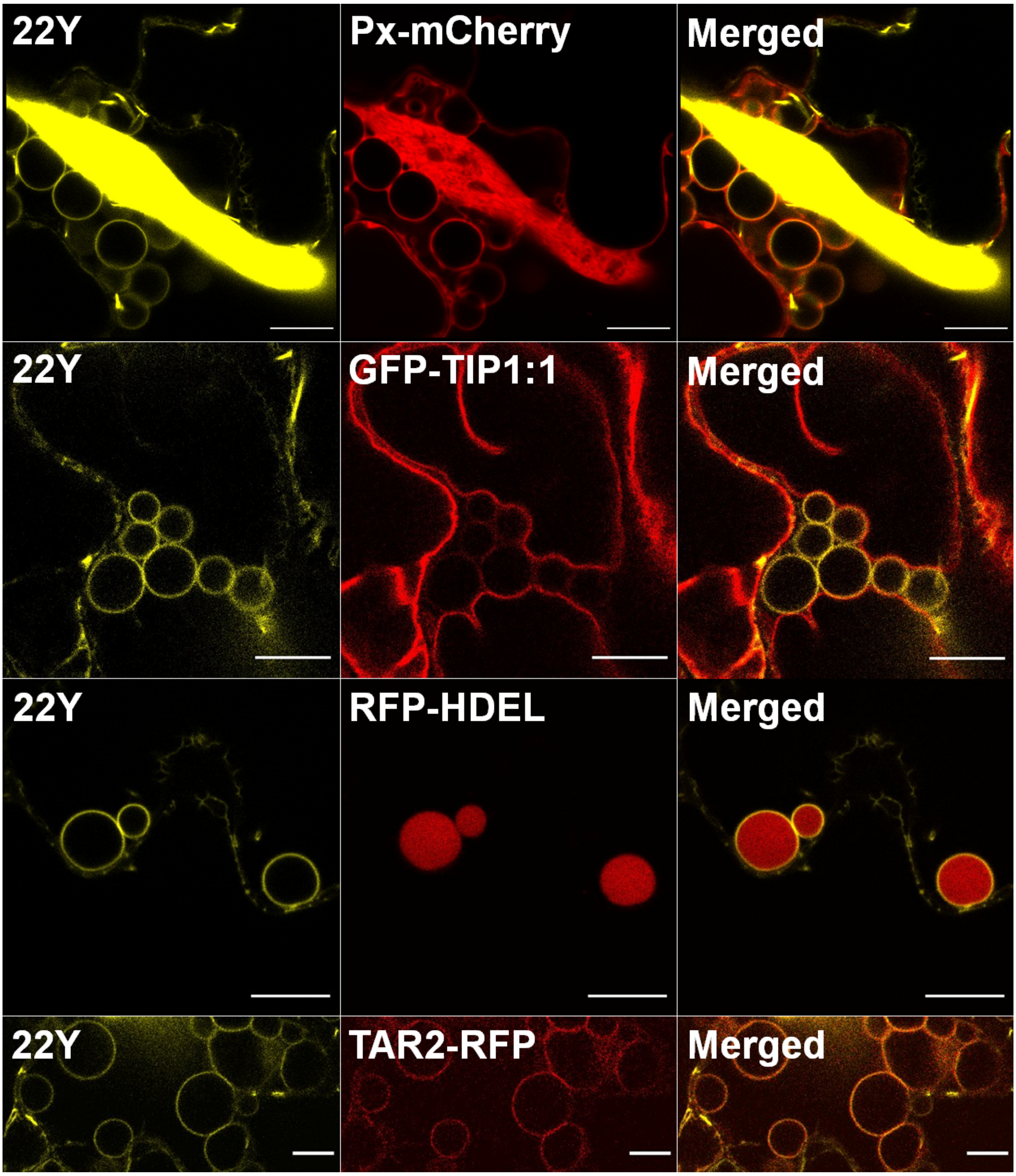

Supplementary Figure S6: Characterisation of the 22Y spheres. Four fluorescent markers were co-expressed with 22Y and visualised 7 days after agroinfiltration of mature *N. tabacum* leaves. The cytosolic Peredox-mCherry image is a high-contrast version of the one from Supplementary Figure S3, showcasing the presence of trapped cytosol next to the 22Y spheres. The tonoplast membrane marker GFP-TIP1:1 is present in the membrane of the spheres, but at lower concentration than in the tonoplast membrane. The luminal ER marker RFP-HDEL is present in the lumen of the spheres, and the ER membrane marker TAR2-RFP is also present in the membrane of the 22Y spheres. Scale bars: 10 µm.

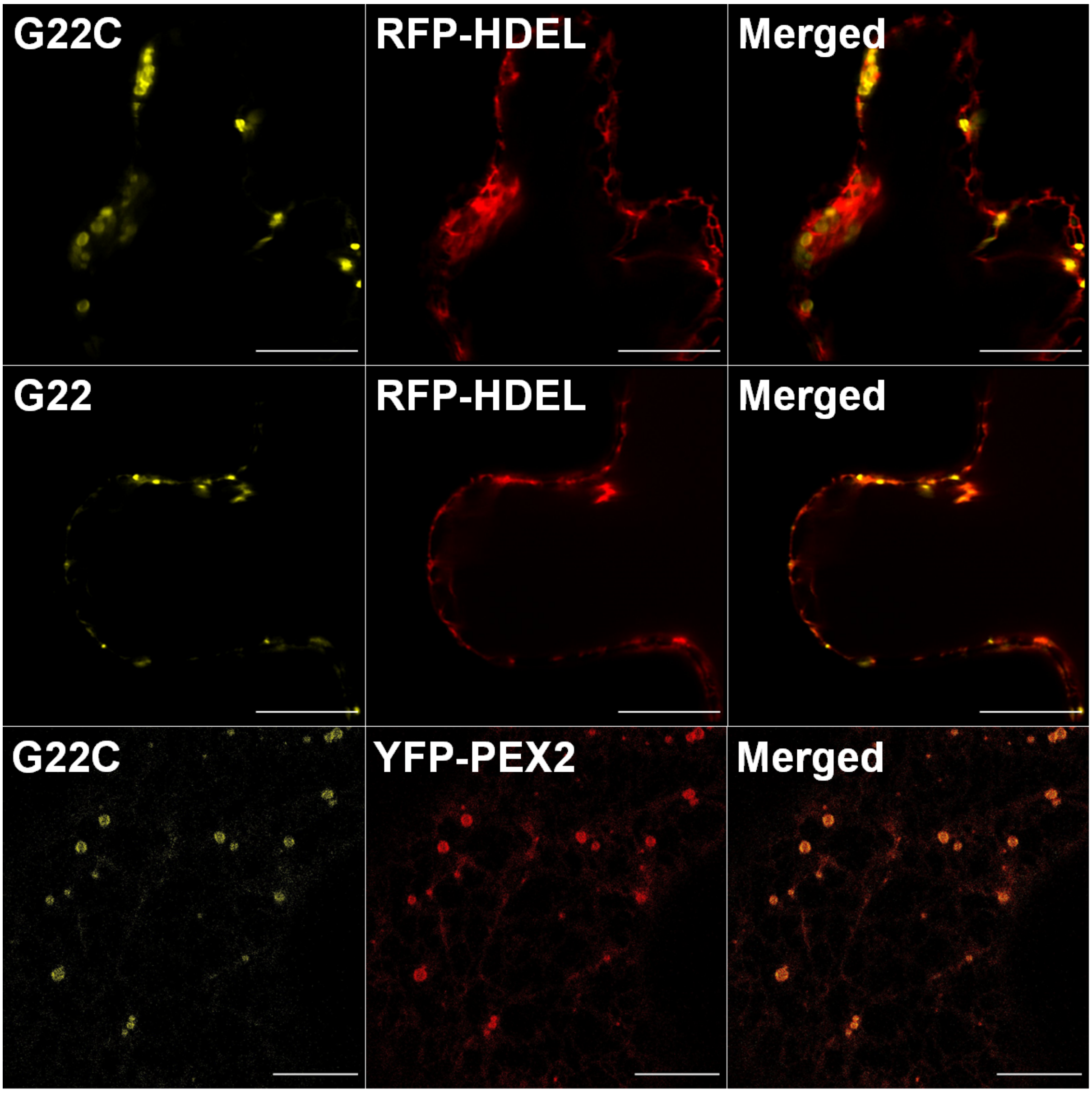

Supplementary Figure S7: Co-expression of fluorescent markers with G22C and G22. RFP-HDEL shows no co-localisation with G22 or G22C signal. Peroxisomal marker YFP-PEX2 co-localises with G22C, suggesting a mis-localisation of the G22C proteins to the peroxisomes, instead of forming the large ER-derived compartment structures. Images were captured 7 days after agroinfiltration of mature *N. tabacum* leaves. Scale bars: 10 µm.

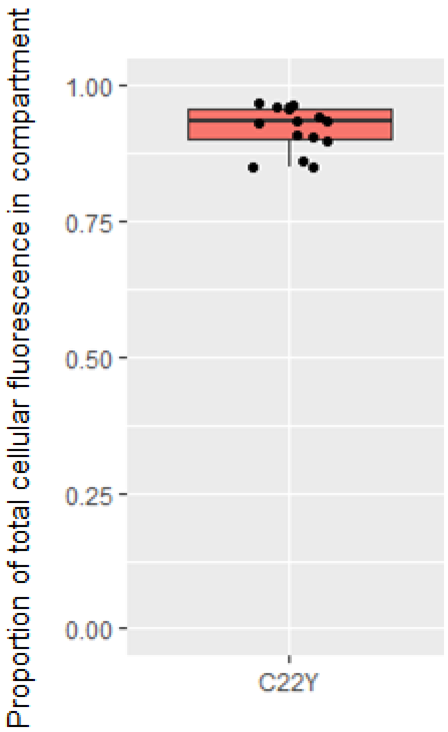

Supplementary Figure S8: Proportion of total cellular fluorescence present in the C22Y compartment. An average of 92.7% of the total cellular YFP fluorescence is localised to the compartment, suggesting that almost all of the C22Y scaffold molecules are present in the compartment. n = 15

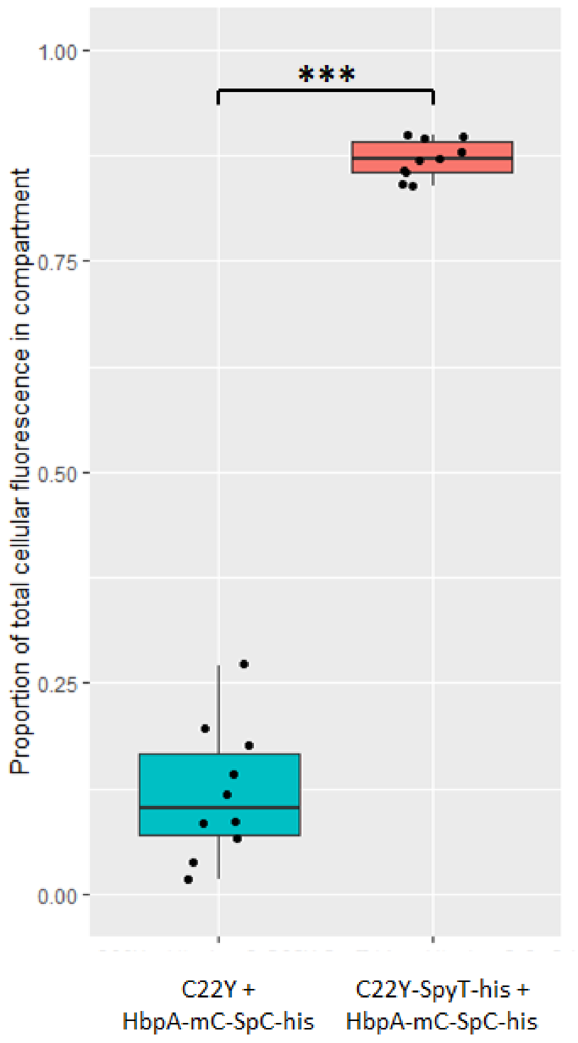

Supplementary Figure S9: Efficient recruitment of proteins to the cytosolic surface of the compartment via SpyCatcher – SpyTag covalent binding. The proportion of total cargo (HbpA-mC-SpC-his) cellular mCherry fluorescence in the compartment. There is a clear statistically significant difference between cargo co-expression with C22Y and C22Y-SpyT-his ($p = 1.3 \times 10^{-16}$). The boxes represent the interquartile range, the horizontal line in the box shows the median, and the whiskers the minimum and maximum values (excluding outliers). n = 10 for both categories

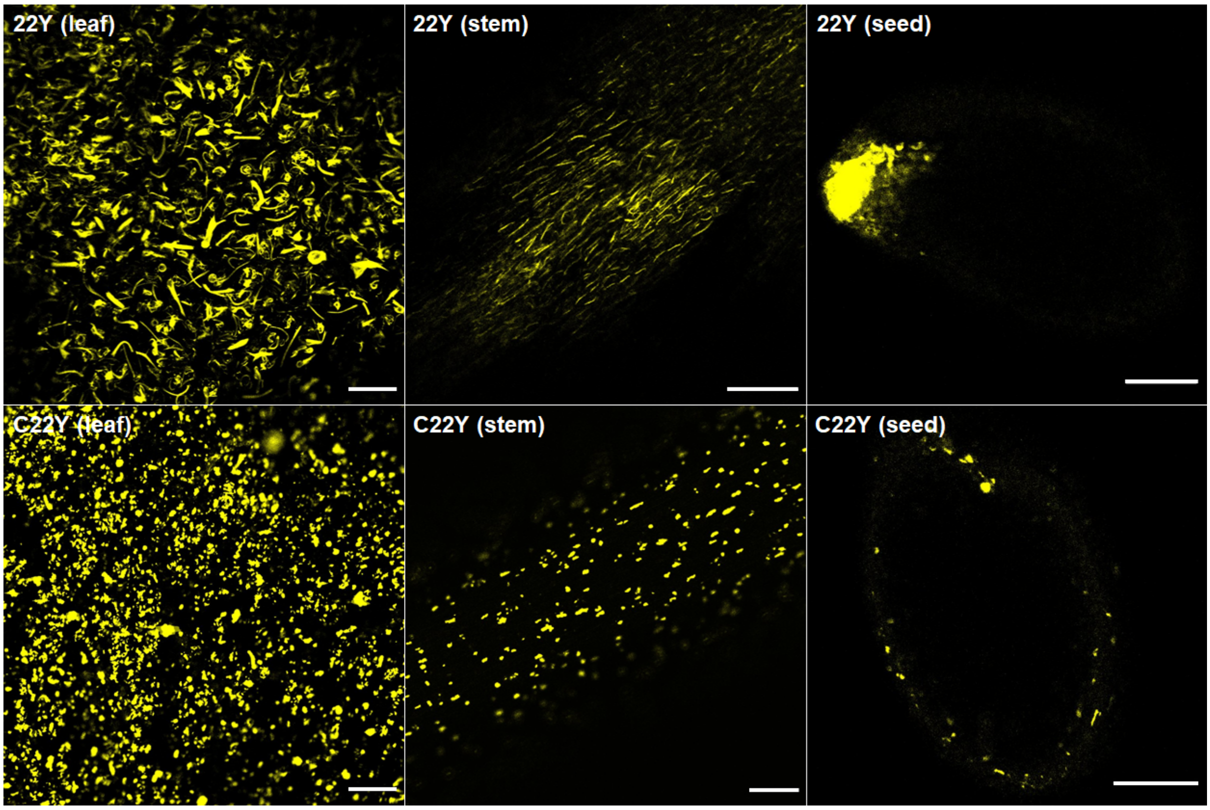

Supplementary Figure S10: Confocal microscopy images of 6 weeks old stably transformed T2 *A. thaliana* lines. YFP fluorescence is consistently present in compartments in all cells for both compartment types. Seeds from both lines were also imaged, with 22Y showing faint fluorescence in the seed coat and most of the signal concentrated in the micropylar endosperm, while C22Y seeds showed several smaller compartment-like structures in the seed coat. Scale bars: 100 µm.

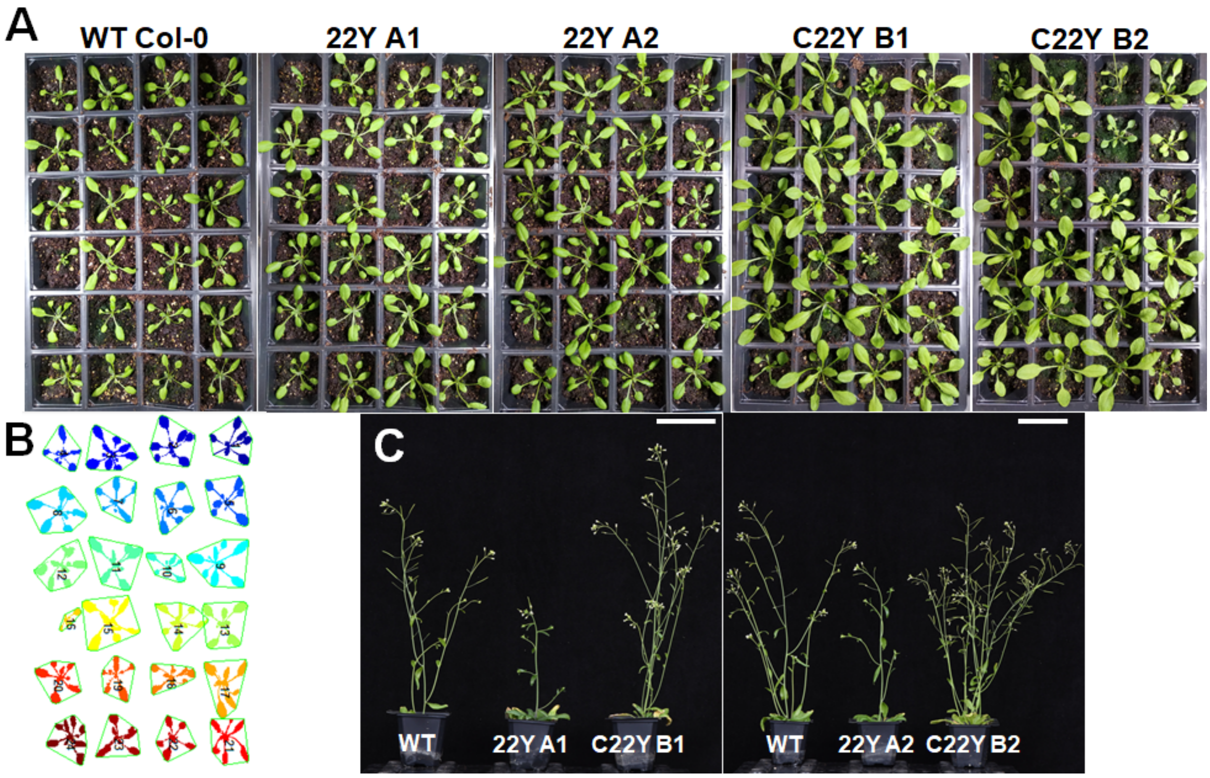

Supplementary Figure S11: Phenotypes of stably transformed *A. thaliana* T2 lines. A: Top-down imaging of T2 lines 3 weeks after transplantation to soil from MS selective media plates. C22Y lines show clearly quicker leaf growth. B: Example image (of WT Col-0 from A) of how the LeafLab image analyser software package differentiates between individual plants. The green outline shows convex area. Solidity is calculated as area (coloured) divided by convex area. C: Side-by-side comparison of 5 weeks old T2 plants. C22Y lines are larger than the WT or 22Y lines, possibly due to the earlier start of stem growth. 22Y showed delayed stalking compared to WT. Scale bar: 5 cm.

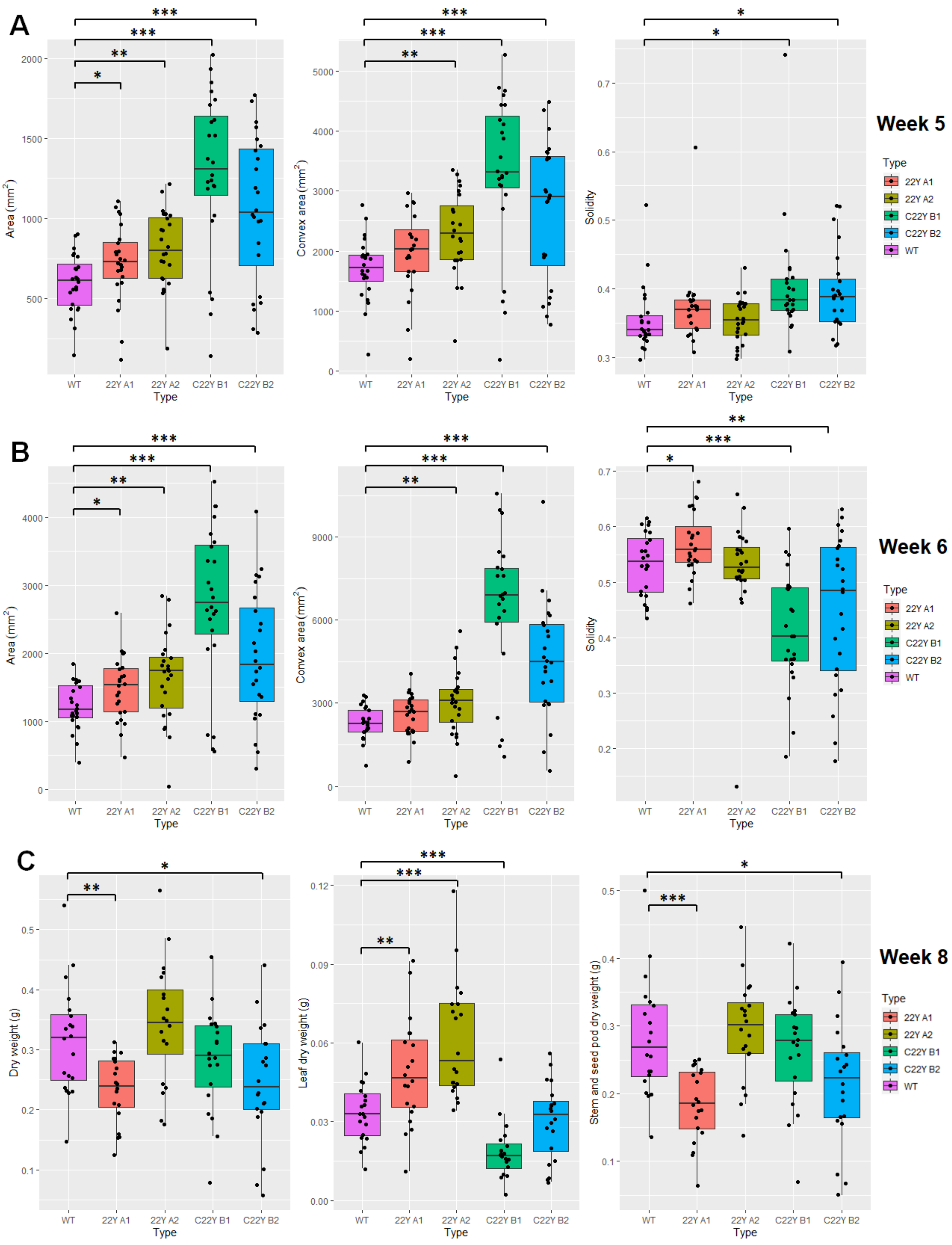

Supplementary Figure S12: Statistical analysis of key macro-phenotypes of stably transformed *A. thaliana* lines. A: Leaf surface area analysis of 4 week old plants (n=24 for each genotype). B: Leaf surface area analysis of 5 week old plants (n=24 for each genotype). Note that by this time, most plants had overlapping leaves, and leaf area at this point is a better estimate of effective photosynthetic leaf area. C: Dry weight measurement of leaves, stem and total above-ground biomass of 8 week old plants (n=20 for each genotype). The boxes represent the interquartile range, the horizontal line in the box shows the median, and the whiskers the minimum and maximum values (excluding outliers).

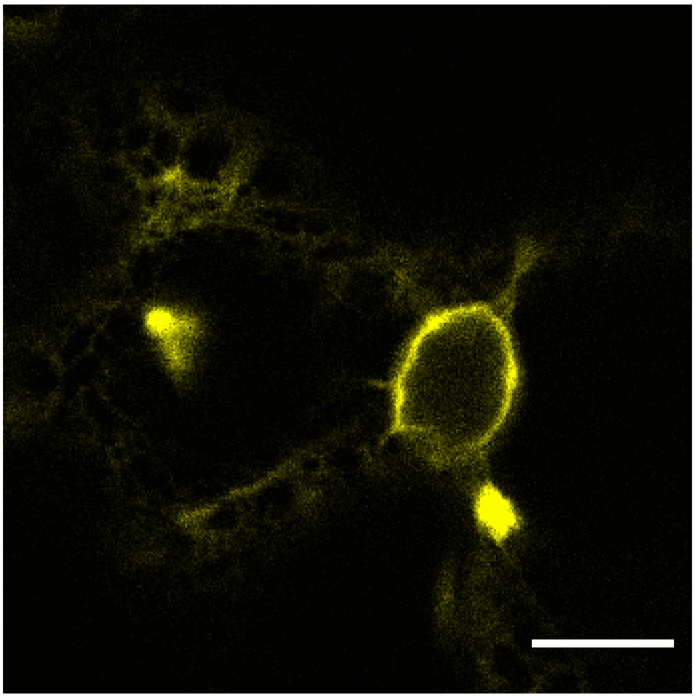

Supplementary Video SV1: ER rearrangements in a cell with a compartment. Oversaturated YFP signal of C22Y compartments were captured to highlight the normal mobility of the ER network. Video was captured 3 days after agroinfiltration of mature *N. tabacum* leaves. Scale bar: 10 µm.

Supplementary Table S1: Parameters used in the AnalyzER software package to investigate ER network dynamics. Descriptions are from the AnalyzER manual (version 1.1). Individual parameters were tested using planned comparisons in ANOVA and one of these was (marked with asterisks) shown to have a statistically significant difference between at least one of the tested compartment types and the WT controls. However, when the ANOVA p-values were corrected for multiple testing, none of the parameters remained significantly different.

Parameter type	Parameter name	Description	ANOVA p-value (uncorrected)
Tubule edges	Length	The total length of tubules (l, µm)	0.932
Tubule edges	Volume	The total cross-sectional volume of tubules ($v = a * l$, µm^3)	0.476
Tubule edges	Resistance	The predicted resistance to flow assuming Poiseuille flow (l/r^4, µm^{-3})	0.746
Tubule edges	Tortuosity	The Euclidean distance between the nodes divided by the total length of the edge	0.578
Tubule edges	Width_center	The estimated width excluding overlap regions at the node	0.574
Tubule edges	Speed_local	The scalar sum of the speeds calculated for each pixel (µm s^{-1})	0.578
Tubule edges	Speed_max	The maximum speed for any pixel (µm s^{-1})	0.546
Tubule edges	Speed_global	The vector sum of speeds for each pixel (µm s^{-1})	0.642
Tubule edges	Flow_coherence	The ratio of the local speed (scalar sum) to the global speed (vector sum)	0.592
Tubule edges	Persistency	The mean period of time that each pixel forms part of a tubule (s)	0.966
Tubule nodes	node_Omin_Omaj	Branch angles between the three main incident tubules, determined from a linear segment from the node to the midpoint of each tubule	0.558
Tubule nodes	node_Omid_Omaj		0.471
Tubule nodes	node_Omin_Omid		0.702
Tubule nodes	node_Speed	Sum of speed at nodes (µm s^{-1})	0.578
Tubule nodes	node_Persistency	The mean period of time that each pixel forms part of a node (s)	0.831
Cisternae	cisternal_Node_degree	The number of connecting tubules incident on the cisterna	0.871
Cisternae	cisternal_Contrast_all	A measure of the intensity contrast between pixel i and its neighbour j. Values range from 0 to (nbins1)^2. Here, results are normalised to (nbins-1)^2 to fall between [0 1]. An idealised cisternal sheet would have a contrast of zero.	0.530
Cisternae	cisternal_Correlation_all	A measure of how correlated a pixel is to its neighbour. where μi and μj are the weighted mean intensities, and σi and σj are the standard deviations of the GLCM distributions. Values range from -1 (un-correlated) to 1 (fully correlated). An idealised cisternal sheet would have a value of 1.	0.368
Cisternae	cisternal_Energy_all	Gives the sum of squared elements in the GLCM. Values range from 0 to 1. An idealised cisternal sheet would have an energy of 0.	0.908
Cisternae	cisternal_Homogeneity_all	Measures the closeness of the distribution of elements in the GLCM to the diagonal. Values range from 0 to 1. An idealised cisternal sheet would have a value of 1	0.373
Cisternae	cisternal_Speed_local	The scalar sum of local speeds at every pixel (µm s^{-1})	0.901
Cisternae	cisternal_Speed_global	The vector sum of global speed (µm s^{-1})	0.379
Cisternae	cisternal_Flow_coherence	The ratio of the local speed (scalar sum) to the global speed (vector sum	0.060

Cisternae	cisternal_MaxPersistency	The maximum persistency of pixels in each cisterna (s)	0.182
Cisternae	cisternal_MeanPersistency	The average persistency of pixels in each cisterna (s)	0.049 (*)
Cisternae	cisternal_MaxDistance	The maximum distance of any pixel to the edge of the cisterna (µm)	0.055
Cisternae	cisternal_MeanDistance	The average distance of any pixel to the edge of the cisterna (µm)	0.063
Cisternae	cisternal_Area	The area of each cisterna	0.129
Cisternae	cisternal_Solidity	The proportion of the pixels in the convex hull that are also in the cisterna	0.129
Cisternae	cisternal_Elongation	The ratio of the major axis to the the minor axis	0.188
Cisternae	cisternal_Roughness	The ratio of the perimeter^2 to the area	0.564
Cisternae	cisternal_circularity	The ratio of the radius determined from the area to the radius determined from the perimeter	0.369
Polygons	polygon_Area	The area of each polygonal region with the ER tubules thinned to a single-pixel wide skeleton	0.316
Polygons	polygon_Solidity	The proportion of the pixels in the convex hull that are also in the region	0.064
Polygons	polygon_Circularity	The ratio of the radius determined from the area to the radius determined from the perimeter	0.079
Polygons	polygon_Roughness	The ratio of the perimeter^2 to the area	0.070
Polygons	polygon_Elongation	The ratio of the major axis to the minor axis	0.816
Polygons	polygon_MaxDistance	The furthest distance within the region to the ER network	0.523
Polygons	polygon_MeanDistance	The average distance within the region to the ER network	0.530
Graph	Geff	The global efficiency of the network	0.411
Graph	Alpha	The alpha coefficient or meshedness	0.842

Supplementary Table S2: Results of statistical analyses of key macro-scale phenotypes. LeafLab was used to determine leaf area, leaf convex area and leaf solidity of T2 lines at week 4 and 5. The dry weight of leaves and stems were measured at week 8. Asterisks show significance (* for $p < 0.05$; ** for $p < 0.01$; *** for $p < 0.001$). n = 24 for week 4 and 5 measurements, n = 20 for dry weight measurements for all categories.

**T-tests against WT (p-values)**	**22Y A1**	**22Y A2**	**C22Y B1**	**C22Y B2**
**Week 4 leaf area**	0.0486 (*)	0.0011 (**)	7.4×10^{-8} (***)	4.6×10^{-5} (***)
**Week 4 leaf convex area**	0.1128	0.0015 (**)	9.2×10^{-7} (***)	0.0005 (***)
**Week 4 leaf solidity**	0.2459	0.9307	0.0130 (*)	0.0121 (*)
**Week 5 leaf area**	0.0304 (*)	0.0065 (**)	1.4×10^{-7} (***)	0.0008 (***)
**Week 5 leaf convex area**	0.1146	0.0055 (**)	1.1×10^{-8} (***)	5.3×10^{-6} (***)
**Week 5 leaf solidity**	0.0314 (*)	0.6458	6.1 x 10-6 (***)	0.0068 (**)
**Total above-ground dry weight**	0.0014 (**)	0.3630	0.2581	0.0240 (*)
**Total leaf dry weight**	0.0059 (**)	3.0×10^{-5} (***)	0.0002 (***)	0.4388
**Total stem dry weight**	0.0001 (***)	0.6512	0.5170	0.0204 (*)

Supplementary Table S3: Gene parts used for the design of the genetic constructs.

Name of gene or fragment	Description	Sequence or accession number
**GFP**	Anti-parallel dimerising green fluorescent protein mGFP5	U87973.1 (European Nucleotide Archive)
**YFP**	Anti-parallel dimerising yellow fluorescent protein eYFP.	AAX97736.1 (GenBank)
**BP22**	Transmembrane domain derived from the BP80 vacuolar sorting receptor of *P. sativum* (Brandizzi *et al.*, 2002)	TTTTGGCTTGCTCTCGTCGTTTTGATCGCCTTAGCTATGATTGCAGGAGGGGATTCCTTGTGTAT
**ER localisation signal**	ER-targeting signal peptide from the original G22Y construct.	AAGACTAATCTTTTTCTCTTTCTCATCTTTTCACTTCTCCTATCATTATCCTCGGCCGAGTTC
**CC-Di**	Parallelly dimerising synthetic coiled coil domain (Fletcher *et al.*, 2012)	GAGATTGCCGCGCTGAAACAGGAGATTGCGGCGCTGAAGAAAGAGAACGCAGCGCTGAAATGGGAGATTGCTGCGTTGAAACAG
**N-Linker**	Linker sequence between BP22 and the ER facing oligomerising domain. Derived from the original G22Y construct.	TCGACTTGGGCTGCT
**C-Linker**	Linker sequence between BP22 and the cytosolic facing oligomerising domain. Derived from the original G22Y construct.	AAATATAGAATTAGGGGAGGAGCTGGAGGTGCTGGCTCGACCATG
**HbpA**	2-hydroxybiphenyl-3-monooxygenase from the 2-2'-biphenol breakdown pathway of *Pseudomonas nitroreducens.*	U73900.1 (GenBank)
**SpyCatcher**	SpyCatcher003 peptide capable of forming a covalent bond *in vivo* with SpyTag003.	GTAACCACCTTATCAGGTTTATCAGGTGAGCAAGGTCCGTCCGGTGATATGACAACTGAAGAAGATAGTGCTACCCATATTAAATTCTCAAAACGTGATGAGGACGGCCGTGAGTTAGCTGGTGCAACTATGGAGTTGCGTGATTCATCTGGTAAAACTATTAGTACATGGATTTCAGATGGACATGTGAAGGATTTCTACCTGTATCCAGGAAAATATACATTTGTCGAAACCGCAGCACCAGACGGTTATGAGGTAGCAACTCCAATTGAATTTACAGTTAATGAGGACGGTCAGGTTACTGTAGATGGTGAAGCAACTGAAGGTGACGCTCATACTGGATCCAGTGGTAGC
**SpyTag**	SpyTag003 peptide capable of forming a covalent bond *in vivo* with SpyCatcher003.	CGTGGCGTGCCTCATATCGTGATGGTGGACGCCTACAAGCGTTACAAG
**mCherry**	Monomeric red fluorescent protein.	AY678264.1 (GenBank)

Supplementary Table S4: PCR and sequencing primers used to generate and confirm constructs.

Primer name	Nucleotide sequence	Description
**Gibson cloning**		
**AS_109F**	CATTATCCTCGGCCGAATTCTCGACTTGGGCTGCTTTTTG	Primers to generate pTWIST-ENTR 22Y from pTWIST-ENTR C22Y
**AS_109R**	CAAAAAGCAGCCCAAGTCGAGAATTCGGCCGAGGATAATG	
**AS_110F**	GAGGTGCTGGCTCGACCATGGAGCAGAAACTTATCTCTGAGGAGG	Primers to generate pTWIST-ENTR G22 from pTWIST-ENTR G22C
**AS_110R**	TCAGAGATAAGTTTCTGCTCCATGGTCGAGCCAGCACCTC	
**AS_203F**	AGTAAAGGAGAAGAACTTTTCAC	Primers to remove ER localisation signal from pTWIST-ENTR G22Y to generate pTWIST-ENTR G-Y(cyt)
**AS_203R**	CATTGTGGACTAGTCTCGAC	
**AS_204F**	GGCTCGACCATGAGCAAG	Primers to remove BP22 transmembrane domain from pTWIST-ENTR G22Y to generate pTWIST-ENTR G-Y(cyt)
**AS_204R**	AGCCCAAGTCGACTTGTATA	
**Sequencing primers**		
**AS_seq_05R**	TTTGTATAGTTCATCCATGCC	Reverse sequencing primer for GFP in G22Y
**AS_seq_06F**	GGAGTACAACTACAACAGCCA	Forward sequencing primer for YFP and BP22 in G22Y
**AS_seq_06R**	CCCTCAGGCATGGCGCTCTTGAA	Reverse sequencing primer for mGFP5 + BP22 in G22Y
**AS_seq_07F**	GCAAAAGAACGGCATCAAAG	Sequencing primers for BP22 in G22Y
**AS_seq_07R**	GTCCAGCTCGACCAGGATGG	
**AS_seq_08F**	TACAAGTCGACTTGGGCTGC	
**AS_seq_08R**	CGGACACGCTGAACTTGTGG	
**AS_seq_09F**	GGAAGTTCATTTCATTCGGAGA	Sequencing primer for G22Y binding upstream of the CaMV35S promoter
**AS_seq_09R**	CATAAATAACGTCATGCATTACATGT	Sequencing primer for G22Y binding in the terminator
**AS_seq_010F**	ATGAAGACTAATCTTTTTCTC	Sequencing primer binding at the ER localisation signal
**AS_seq_09F_alt**	GGAAGTTCATTTCATTTGGAGA	Sequencing primer for pK7WG2 binding in the p35S

**AS_seq_011R**	AGGTCACTGGATTTTGGTTTTAG	Sequencing primer for pK7WG2 binding in the T35S
**AS_seq_M13F**	GTAAAACGACGGCCAGT	Common sequencing primer M13 forward for pTWIST-ENTR
**AS_seq_M13R**	CAGGAAACAGCTATGAC	Common sequencing primer M13 reverse for pTWIST-ENTR
